# The non-classical major histocompatibility complex II protein SLA-DM is crucial for African swine fever virus replication

**DOI:** 10.1038/s41598-023-36788-9

**Published:** 2023-08-21

**Authors:** Katrin Pannhorst, Jolene Carlson, Julia E. Hölper, Finn Grey, John Kenneth Baillie, Dirk Höper, Elisabeth Wöhnke, Kati Franzke, Axel Karger, Walter Fuchs, Thomas C. Mettenleiter

**Affiliations:** 1 Institute of Molecular Virology and Cell Biology, Friedrich-Loeffler-Institut, Südufer 10, 17493 Greifswald-Insel Riems, Germany; 2 The Roslin Institute, University of Edinburgh, Midlothian, UK; 3 Institute of Diagnostic Virology, Friedrich-Loeffler-Institut, Greifswald-Insel Riems, Germany; 4 Institute of Infectology, Friedrich-Loeffler-Institut, Greifswald-Insel Riems, Germany; 5 Friedrich-Loeffler-Institut, Greifswald-Insel Riems, Germany; 6 Present Address: Ceva Animal Health, Greifswald-Insel Riems, Germany

**Keywords:** Virus-host interactions, High-throughput screening

## Abstract

African swine fever virus (ASFV) is a lethal animal pathogen that enters its host cells through endocytosis. So far, host factors specifically required for ASFV replication have been barely identified. In this study a genome-wide CRISPR/Cas9 knockout screen in porcine cells indicated that the genes *RFXANK, RFXAP, SLA-DMA, SLA-DMB,* and *CIITA* are important for productive ASFV infection. The proteins encoded by these genes belong to the major histocompatibility complex II (MHC II), or swine leucocyte antigen complex II (SLA II). RFXAP and CIITA are MHC II-specific transcription factors, whereas SLA-DMA/B are subunits of the non-classical MHC II molecule SLA-DM. Targeted knockout of either of these genes led to severe replication defects of different ASFV isolates, reflected by substantially reduced plating efficiency, cell-to-cell spread, progeny virus titers and viral DNA replication. Transgene-based reconstitution of SLA-DMA/B fully restored the replication capacity demonstrating that SLA-DM, which resides in late endosomes, plays a crucial role during early steps of ASFV infection.

## Introduction

African swine fever virus (ASFV) is the causative agent of African swine fever (ASF), a hemorrhagic disease of domestic pigs and wild boar (species *Sus scrofa*)^1–3^. ASFV was first identified in Kenya in 1921, and has been reported since then in most sub-Saharan African countries^4,5^. By partial sequencing of the gene B646L, which codes for the major capsid protein p72, twenty-four genotypes of ASFV have been specified^6,7^. Up to now, only two of them, genotype I and genotype II, have been detected outside Africa, with genotype II being responsible for the current panzootic. Since the introduction of ASFV into Georgia in 2007 outbreaks of ASF have been reported in countries of the European region, the Russian federation, Asia, Oceania and the Americas^3,8,9^ (OIE-Wahis Website, visited November, 2022). In most affected countries the control of ASF is still limited to biorisk management measures, surveillance approaches and response strategies, which include culling and trade restrictions, and result in significant economic and production losses, as licensed vaccines are currently not commercially available^3^.

ASFV is the only member of the genus *Asfivirus* of the family *Asfarviridae*^10–13^. Its linear double stranded DNA genome varies between 170 and 193 kbp in size and contains 150 to 167 predicted protein-encoding open reading frames^14–16^. The existence of 134 viral proteins has been confirmed, but for many of them functions are still unknown, or could only be predicted based on sequence homologies^17–21^. ASFV particles contain approximately 82 viral proteins, are about 250 nm in size and multilayered. They comprise an external lipid membrane, an icosahedral outer capsid, an internal lipid membrane, an inner capsid, a thick protein core shell, and a nucleoid containing the genome^18,19,22,23^.

ASFV particles enter their host cell either through dynamin- and clathrin-mediated endocytosis^24,25^ or through macropinocytosis^26,27^. Once ASFV particles have been internalized they traffic through the entire endolysosomal pathway. Directly after infection they can be detected in early endosomes or macropinosomes by electron microscopy and by colocalization of viral proteins with specific endosomal markers (EEA1 and Rab5)^26^. At later time points ASFV is found in late endosomes or lysosomes colocalizing with CD63, Rab7, Lamp1 and cathepsin^26,28^. During this transport, ASFV particles undergo extensive structural changes that result in virions lacking outer membranes and outer capsids within multivesicular late endosomes. The exposed inner envelope of ASFV particles then merges with the endosomal membrane resulting in release of naked core particles into the cytoplasm^26^. Following a short and poorly understood initiation step within the nucleus, viral DNA replication and virus particle morphogenesis take place within the cytoplasm at perinuclear virus factories adjacent to the microtubule organizing center^29^. For the formation of progeny virus particles, membranes derived from the endoplasmic reticulum (ER) are acquired, and intracellular virions consisting of the genome containing inner core, inner capsid, inner envelope, and outer capsid are assembled^30–33^. These intracellular virions are then transported along microtubules to the cell periphery and acquire their external lipid membrane by budding from the plasma membrane^34–36^. Both the intracellular virions as well as doubly enveloped virions are infectious^32^.

As an obligate intracellular pathogen ASFV depends on host cell metabolism and proteins for its replication. The entry process of ASFV for example is temperature, energy and cholesterol dependent ^37,38^. It requires the activity of dynamin, Rac1, Pak1, Rab7, endosomal membranes with phosphoinositides synthesized by phosphatidylinositol kinases (PI3K or PIKfyve), and the acidification of the endosomes^25–28,39–41^. In addition, it has been shown that the abrogation of the endosomal protein Niemann Pick C1 (NPC1) or the lysosomal membrane protein 1 (Lamp-1), partially inhibited ASFV infection. As a complete inhibition was not achieved, it was speculated that additional endosomal proteins may play a role during this step of ASFV replication^42^.

The CRISPR/Cas9 technology offers methods to identify host–pathogen interactions in unbiased high throughput approaches^43^. In the past, this technology was already used to identify host factors relevant for replication of e.g. influenza virus, flavivirus, norovirus, Schmallenberg virus, human immunodeficiency virus, hepatitis C virus, and Japanese encephalitis virus^44–51^. Moreover, using a newly developed lentivirus-based CRISPR/Cas9 library targeting the porcine genome (SsCRISPRko.v1), a novel host factor required for replication of the porcine alphaherpesvirus pseudorabies virus (PrV) was identified^52^.

In this study the CRISPR-knockout single guide RNA (sgRNA) library which is targeting all annotated genes of the porcine (*Sus scrofa*) genome was used for a genome-wide screen to identify cellular factors which are relevant for ASFV replication. Our studies discovered that knockout of five expression factors and membrane proteins of the major histocompatibility complex II (MHC II), also called swine leucocyte antigen complex II (SLA II), almost abrogates reproductive ASFV infection. In particular, the heterodimeric non-classical MHC II protein SLA-DM was found to be crucial for ASFV replication.

## Results

### A genome-wide CRISPR/Cas9 knockout screen identified cellular factors of the MHC II pathway relevant for ASFV replication

To identify host genes and their respective protein products that are required for ASFV replication in cultured porcine cells, a genome-wide CRISPR/Cas9 screen was performed with the porcine CRISPR/Cas9 knockout library SsCRISPRko.v1 which was previously described and characterized^52^. The library encoded 83,381 sgRNAs encompassing 1001 non-targeting control sgRNAs, and 82,380 specific sgRNAs targeting 20,598 porcine genes with three to four sgRNAs per gene. The sgRNA sequences were cloned into the vector lentiCRISPRv2, which also provides an expression cassette for Cas9, and a puromycin resistance gene for selection.

This library was packed into defective lentivirus particles which were then used to transduce highly passaged wild boar lung (WSL) cells, which support efficient replication of many native or adapted ASFV isolates^53,54^. To ensure the integration of only one sgRNA gene into the genome of a single cell, a low multiplicity of transduction (MOT) of 0.3 was chosen. Puromycin resistant cells that putatively stably expressed Cas9 and single sgRNAs were expanded over two weeks. At that point, about 6 × 10^7^ cells of the total number of approximately 4 × 10^8^ cells per experiment were stored as uninfected controls for DNA preparation. The remaining cells were reseeded and infected with genotype IX recombinant ASFV Kenya 1033 ΔCD2v dsRed^55,56^ at a multiplicity of infection (MOI) of 0.3 or 0.5. The fluorescent expression marker facilitated the detection of successful infection. A progressive cytopathic effect (CPE) was detectable from 48 h after infection, and after approx. four to five weeks cell colonies became visible that originated from single surviving cells. These cells were pooled in two subsets. Parts of these pools were saved as ‘survivors 1–1’ and ‘survivors 1–2’ for DNA preparation. The other parts were reseeded and infected again, and after approx. three weeks ‘survivors 2–1’ and ‘survivors 2–2’ could be harvested. Seeding and infection of the cells was repeated four times to ensure exposure of all cells to the virus. The repeated infections were necessary, as it was noticed that ASFV Kenya, although inducing a very pronounced CPE in WSL cells, was not always able to lyse all non-transduced control cells during one round of replication. To further exclude false hits from accidentally surviving cells, the screening procedure was performed not only with two cell subsets, but also in two independent experiments.

For each screen the DNA of the control (cells before infection) and of the survivors (cells after the infection) of the two subsets was isolated in parallel and the integrated sgRNA gene regions were amplified in three sequential PCRs with suitable primers (Supplementary Table S1) for Ion Torrent sequencing. Sequencing data was analyzed using the MAGeCK algorithm software which tests whether the abundance of sgRNA genes differs significantly between treated cells (survivors) and controls, and identifies enriched sgRNA sequences targeting specific genomic loci with the calculation of the robust ranking aggregation (RRA)^57^.

**Figure 1:**
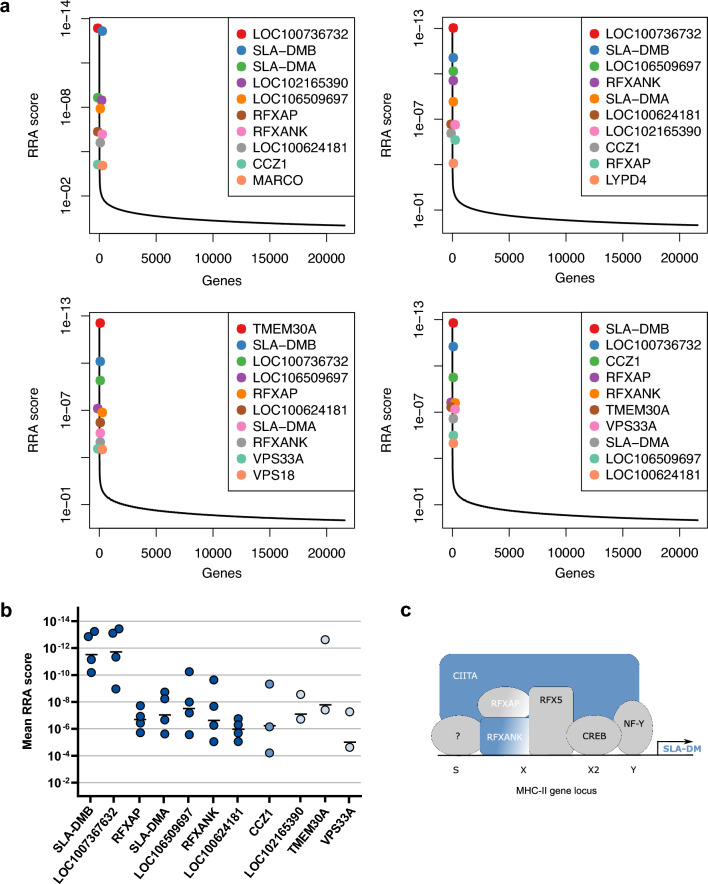
Genome-wide CRISPR/Cas9 knockout screens identified molecules of the MHC II pathway as relevant for ASFV replication. (**a**) Diagrams of robust rank aggregation (RRA) scores calculated by the MAGeCK algorithm software of four separate analyses determined in two independent screens. The sgRNA content of the control cells was compared to the sgRNA abundance of cells that survived four subsequent ASFV infections. (**b**) Mean (−) and single RRA scores of the individual gene hits in the four different analyses. Dark blue dots represent sgRNAs against the indicated genes (x-axis) found in 4/4 subsets. Medium light blue dots represent the sgRNAs against gene CCZ1 which were found in 3/4 subsets. Light blue dots show sgRNAs which were only found in the 2 subsets of either of the performed screens. (**c**) Schematic of an MHC II gene locus (e.g. for SLA-DMA or SLA-DMB) with the MHC class II specific regulatory SXY module and specific transcription factors. Proteins which were identified as crucial cellular factor for ASFV infection in the genome-wide CRISPR/Cas9 knockout screen are shown in blue.

With this analysis the sgRNAs targeting *SLA-DMB, LOC100736732, RFXAP, SLA-DMA, LOC106509697, RFXANK,* and *LOC100624181* were found with lowest RRA scores (i.e. most elevated) among the best ten hits of the positively selected genes in all four subsets of the two screens (Fig. 1a, b, Supplementary Table S2). For all of these genes more than one specific sgRNA was found elevated in the surviving cell pool (Supplementary Tables S2 and S3). In addition, the gene *CCZ1* from the best ten hits of the screens was identified in three subsets, whereas the genes *LOC102165390, TMEM30A,* and *VPS33A* were found in one of the screens, and the genes *MARCO*, *LYPD4*, and *VPS18* were under the first ten hits in only one of the subsets (Fig. 1a, b, Supplementary Tables S2 and S3). The protein products of most of the latter genes are involved in endocytosis and/or autophagy pathways, and will be further analyzed in future studies.

This study focuses on the role of host genes that were identified in all four subsets with very low scores (*SLA-DMB, LOC100736732, RFXAP, SLA-DMA, LOC106509697, RFXANK,* and *LOC100624181*). Remarkably, all of them are related to the major histocompatibility complex II (MHC II/SLA II) (Fig. 1c). *RFXANK* codes for the regulatory factor X associated ankyrin containing protein (RFXANK), and *RFXAP* encodes the regulatory factor X associated protein (RFXAP). RFXANK, RFXAP and the regulatory factor X 5 (RFX5) assemble and bind to the X box of the SXY module of MHC II gene promoters (Fig. 1c). Together with other factors they act as landing pad for the MHC class II transactivator (CIITA)^58^. CIITA was identified by elevated sgRNAs against *LOC100736732, LOC106509697,* and *LOC100624181.* RFXAP, RFXANK and CIITA are highly important for transcriptional activity of MHC class II promoters^58^. Lastly, the genes *SLA-DMA* and *SLA-DMB* were identified in both screens which code for the alpha and beta chains of the non-classical class II swine leucocyte antigen DM (SLA-DM), and are transcribed with the help of the above-mentioned factors. In summary, several lines of evidence point to the MHC II pathway as highly relevant host factor for ASFV replication.

### Generation of targeted SLA-DMA, SLA-DMB, RFXAP and CIITA knockout cell lines

To verify the importance of the MHC II expression and presentation pathway for the replication cycle of ASFV, targeted gene knockouts were introduced into a newly isolated single cell clone of the WSL cell line. A clone of WSL cells was used to minimize the risk that results might be affected by inherent genetic differences. For the targeted knockout, the genes encoding the non-classical MHC II molecule SLA-DM, *SLA-DMA* and *SLA-DMB*, as well as two genes that are important for the transcription of MHC II, *RFXAP* and *CIITA* (*LOC100736732*)*,* were selected.

For the knockout, one of the four pig library sgRNA sequences was selected (Supplementary Fig. S1, dark blue; Supplementary Table S4), and cloned into the sgRNA and Cas9 double expression vector pX330A-1 × 4neoRA. WSL cells were transfected with the obtained plasmids, serially diluted and selected for G418 resistance. Resistant single cell clones were propagated and checked for Cas9 expression by immunoblotting. DNA of Cas9 positive cells was then isolated and correct sgRNA gene integration was verified by PCR amplification and sequencing with suitable primers (Supplementary Table S5). Furthermore, PCR analyses with primers specific for the targeted gene regions of *SLA-DMA, SLA-DMB, RFXAP,* and *CIITA* (Supplementary Table S5) were performed. After sequence analysis of the amplification products, WSL knockout (WSL_KO_) cell clones with deleterious nucleotide insertions or deletions (INDELs) in *SLA-DMA* (clones 11, 12, 16), *SLA-DMB* (clones 9, 16, 18), *CIITA* (clones 1, 4, 8), or *RFXAP* (clones 6, 8) were selected (Supplementary Fig. S2). The cell clone WSL SLA-DMA_KO_ (11) displayed an 823 nt insertion containing stop codons in all reading frames, which is derived from the transfer vector pX330A-1×4neoRA. Clones WSL SLA-DMA_KO_ (12) and (16), as well as WSL SLA-DMB_KO_ (9) possess deletions of 1 nt leading to frameshifts and premature stop codons (Supplementary Fig. S2). In contrast, WSL SLA-DMB_KO_ (16) and (18) exhibit an identical insertion of 1 nt (T) which directly creates a stop codon (TGA). The cell clones WSL CIITA_KO_ (1), (4), and (8) displayed deletions of 1, 5, and 23 nucleotides, respectively, leading to frameshifts and premature termination. WSL RFXAP_KO_ (6) exhibited an insertion of 1 nt (C), leading to a downstream termination codon (TGA), and in WSL RFXAP_KO_ (8) a stop codon-containing insertion of 140 nt from the vector pX330A-1×4neoRA was found (Supplementary Fig. S2). Remarkably, with all selected cell clones only single PCR products exhibiting unambiguous sequences of the mutated genes were obtained, indicating either identical biallelic changes or large deletions in the other alleles, which included the primer binding sites. Wildtype sequences of the sgRNA target regions were never observed.

**Figure 2:**
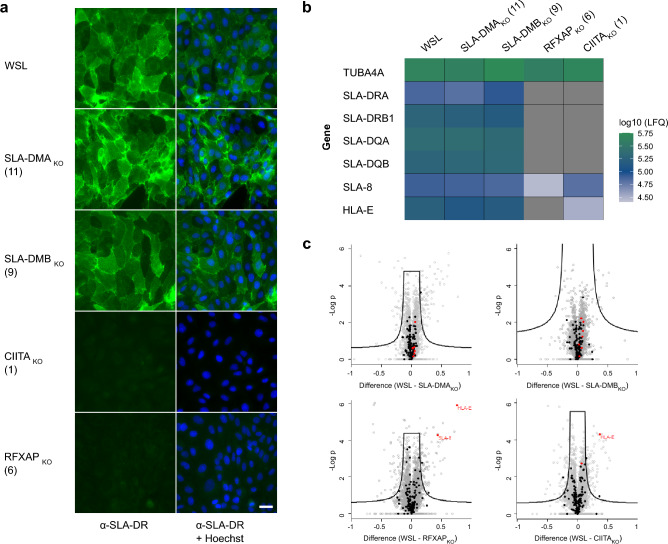
Parental WSL and WSL knockout cells differ in the expression of MHC II proteins. (**a**) Indirect immunofluorescence analyses of cell surface expression of SLA-DR in WSL, WSL SLA-DMA_KO_, WSL SLA-DMB_KO,_ WSL CIITA_KO_ and WSL RFXAP_KO_ cell clones. Bar: 30 µm. (**b**) Mass spectrometry analysis of quantitative expression levels of the housekeeping gene α-tubulin (TUBA4A-ENSSSCG00000016216), of genes of the MHC II pathway (SLA-DRA-ENSSSCG00000001453, SLA-DRB1-ENSSSCG00000001455, SLA-DQA-ENSSSCG00000001456, SLA-DQB-ENSSSCG00000001457), and of the MHC I pathway (SLA-8-ENSSSCG00000001231, HLA-E-ENSSSCG00000001229) in WSL and indicated knockout cells based on label-free quantitation (LFQ). Data represent means of three replicates. Grey panels indicate that the respective proteins were not detected. (**c**) Comparative quantitative analysis of protein expression levels in WSL and individual WSL_KO_ cell clones. Proteins are indicated by dots. Black dots represent proteins involved in antigen processing and presentation. As far as detected, the SLA I/II proteins shown in b are highlighted in red.

Previous studies indicated that WSL cells express the MHC II protein SLA-DR at their surface^54^. This was verified by indirect immunofluorescence (IF) analyses of non-permeabilized cells using an SLA-DR specific monoclonal antibody (mAb) (Fig. 2a). Whereas SLA-DR was detectable on the surface of parental WSL cells and the knockout cells WSL SLA-DMA_KO_ and WSL SLA-DMB_KO_, it was not visible on RFXAP and CIITA knockout cells (Fig. 2a). This confirmed that RFXAP and CIITA are essential factors for MHC II transcription.

For the characterization of the knockouts on proteome level the protein contents of the knockout cell lines were analyzed by mass spectrometry (MS) (Fig. 2b, c). Of the 5495 proteins that were identified and quantified, 4874 were detected in all cell clones (Supplementary Table S6). The protein composition of all cell clones was very similar, as indicated by the lack of a clear clustering of the replicates after principal component analysis (PCA) (Supplementary Fig. S3). Unfortunately, SLA-DM expression was not detectable in the MS analyses, neither in knockout nor in normal WSL cells. However, single knockout of RFXAP or CIITA suppressed the synthesis of other identified MHC II proteins (SLA-DR, SLA-DQ) below the detection levels, and also affected the expression of MHC I (SLA-8, HLA-E), while the expression of these proteins remained unaffected in WSL SLA-DMA_KO_ and WSL SLA-DMB_KO_ cells (Fig. 2b, c).

### SLA-DM, RFXAP and CIITA are required for efficient ASFV replication in WSL cells

Before testing whether ASFV is able to replicate in cells lacking molecules of the MHC II pathway, unspecific side effects of these knockouts on viability and susceptibility to other virus infections should be excluded. For this purpose, parental WSL, WSL SLA-DMA_KO_ (clones 11, 12, 16), WSL SLA-DMB_KO_ (clones 9, 16, 18), WSL CIITA_KO_ (clones 1, 4, 8), and WSL RFXAP_KO_ (clones 6, 8) cells were infected with a GFP-expressing mutant of the porcine alphaherpesvirus pseudorabies virus (PrV). The studies revealed that neither plating efficiency, nor plaque sizes, or progeny virus titers of PrV differed significantly between parental WSL cells and the tested knockout cell lines (Supplementary Fig. S4). This demonstrated that the knockout cells are per se suitable for propagation of a porcine virus.

**Figure 3:**
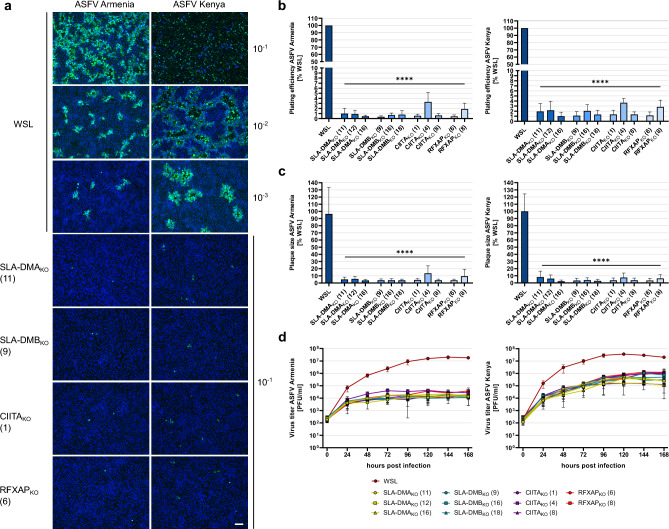
ASFV replication in WSL knockout cells is impaired. (**a**) Visualization of ASFV Armenia or ASFV Kenya-infected WSL and WSL_KO_ cells (green) and nucleic acids (blue) by immunofluorescence staining. Representative images of the indicated cell clones infected with different virus dilutions (10^–1^ to 10^–3^) to illustrate plating efficiency and plaque sizes. Bar: 100 µm. **(b)** Plating efficiency of ASFV Armenia and ASFV Kenya was calculated by counting ASFV-infected cells or plaques in three independent experiments (n = 3). Mean relative apparent titers (%) compared to those on WSL cells and standard deviations are shown. Significant differences were calculated by ordinary one-way ANOVA followed by Tukey’s multiple comparison test. **** = p < 0.0001. (**c**) For the determination of plaque sizes, areas of fifty plaques per cell line from three independent experiments (n = 150) were measured, and the mean relative areas (%) compared to WSL cells including standard deviations are shown. Significant differences were calculated by Kruskal–Wallis test followed by Dunn’s multiple comparison test. **** = p < 0.0001. (**d**) Multi step (MOI 0.02) growth curve analysis of ASFV Armenia or Kenya in WSL and WSL_KO_ cells. Shown are the mean results of three independent experiments (n = 3) with standard deviations.

For ASFV infection of the cells two different virus strains were used. Besides the parental genotype IX strain of the virus recombinant used for library screening (ASFV Kenya 1033), a variant of the current panzootic genotype II virus was also included (ASFV Armenia 2008). Parental WSL and knockout cell clones were infected with serial dilutions of both viruses. After 4 days under semi-solid medium the cells were fixed and infected cells and virus plaques were visualized by IF detection of the ASFV capsid protein p72. Fluorescence microscopy revealed that both strains were able to infect considerably more parental WSL cells than any of the knockout cells (Fig. 3a). The calculated plating efficiencies on all tested WSL_KO_ cells were significantly reduced to < 4% compared to WSL cells (Fig. 3b). In addition, plaque areas were significantly reduced to less than 14% for ASFV Armenia and less than 10% for ASFV Kenya in WSL_KO_ cells compared to plaque sizes of the respective virus on the parental cell line (Fig. 3c). A serious impairment of ASFV replication was also seen in multistep (MOI 0.02) growth studies. Whereas ASFV Armenia replicated in WSL cells to maximum titers of 1.8 × 10^7^ PFU/ml at 168 h post infection (p.i.), the titers in knockout cells ranged from 1.2 × 10^4^ PFU/ml in WSL SLA-DMB_KO_ (18) to 3.8 × 10^4^ PFU/ml in WSL RFXAP_KO_ (8) (Fig. 3d). The final titer of ASFV Kenya in WSL cells was 2.0 × 10^7^ PFU/ml, whereas in knockout cells the titers ranged from 1.2 × 10^5^ PFU/ml in WSL SLA-DMA_KO_ (16) to 1.23 × 10^6^ PFU/ml in WSL RFXAP_KO_ (8) cells (Fig. 3d). Thus, the titers for ASFV Armenia were decreased by 3 logs, and the titers of ASFV Kenya were reduced by at least 1.5 logs on WSL knockout compared to parental cells. Moreover, it was apparent that productive ASFV Armenia replication in knockout cells reached a plateau at 72 h p.i., while the titers of ASFV Kenya increased until 120 h p.i. This might indicate that the few knockout cells successfully infected with ASFV Kenya are able to produce more infectious virus for a longer period of time than ASFV Armenia infected cells, which would be in line with slightly higher titers of ASFV Kenya observed on normal WSL cells.

**Figure 4:**
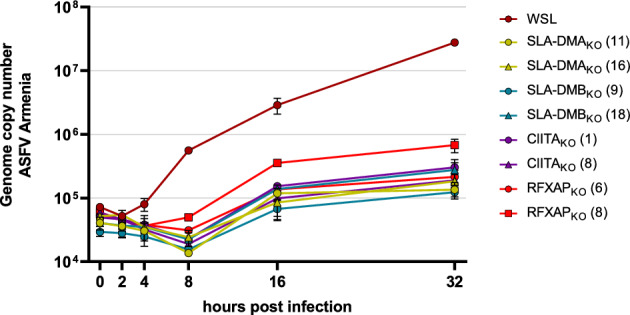
ASFV DNA replication is inhibited in WSL knockout cells. Parental WSL and WSL_KO_ cells were infected with ASFV Armenia at a MOI of 3 and after indicated times the amounts of ASFV DNA were quantified by real-time qPCR targeting the viral B646L gene. Genome copy numbers were determined using plasmid standards. Graphs represent means of two biological replicates with standard deviations.

In addition to the analysis of infectious virus progeny in infected cell lysates, viral DNA replication was also investigated (Fig. 4). To this end, parental WSL cells and selected WSL_KO_ cell clones were infected with ASFV Armenia at a MOI of 3 and harvested at 0, 2, 4, 8, 16 and 32 h p.i. Total DNA was prepared and used to determine ASFV genome copy numbers by duplex TaqMan qPCR reactions for the detection of the viral B646L gene and the host cell β-actin gene as an internal control (Fig. 4). B646L specific probes showed moderately increased DNA amounts after 4 h, and an exponential replication phase until 8 h p.i. in parental WSL cells. At later times, viral DNA replication slowed and resulted in 2.8 × 10^7^ genome copies in the analyzed samples (containing DNA of approx. 1 × 10^4^ cells) at 32 h p.i. A delayed onset of DNA replication was seen in all WSL_KO_ cell clones resulting in a moderate increase of genome copy numbers between 8 h p.i. and 16 h p.i. From 16 h p.i. until the end of the experiment the viral DNA amount remained almost constant and ranged from 1.2 × 10^5^ ASFV genomes in SLA-DMB_KO_ (9) cells to 6.8 × 10^5^ ASFV genomes in RFXAP_KO_ (8) cells. Since the ASFV genome copy numbers were considerably lower in all tested WSL_KO_ cells at all analyzed time points the deleted host proteins are obviously important for a step preceding the onset of viral genome replication.

**Figure 5:**
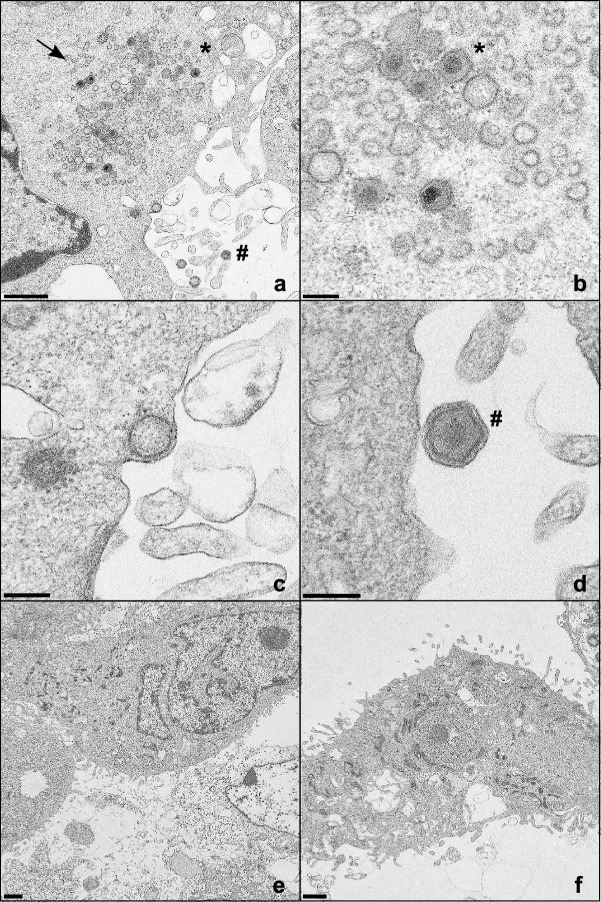
ASFV progeny virus particles are detected in infected parental WSL cells but not in knockout cells. (**a**–**d**) WSL, (**e**) WSL SLA-DMA_KO_, and (**f**) WSL CIITA_KO_ cells were fixed and analyzed by electron microscopy 16 h after infection with ASFV Armenia at a MOI of 5. Virus factories (arrow), intracellular (*) and extracellular virus particles (#) are indicated. Bars represent 1 µm (**a, e, f**), or 200 nm (**b, c, d**).

To further elucidate which steps of virus replication cycle might be blocked after knockout of the MHC II related genes, WSL, WSL SLA-DMA_KO_ (11), and WSL CIITA_KO_ (1) cells were analyzed by electron microscopy (EM) 16 h after infection with ASFV Armenia at a MOI of 5 (Fig. 5). Mature extracellular virus particles, intracellular particles and virus factories were only detected in parental WSL cells, whereas no traces of ASFV replication were found in either of the knockout cells. These results also indicate a function of the knocked-out host cell proteins in initial steps of virus replication, which is in line with the substantially reduced plating efficiency and genome copy number of ASFV in WSL_KO_ cells.

### Susceptibility to ASFV infection can be restored by reintroduction of SLA-DM

**Figure 6:**
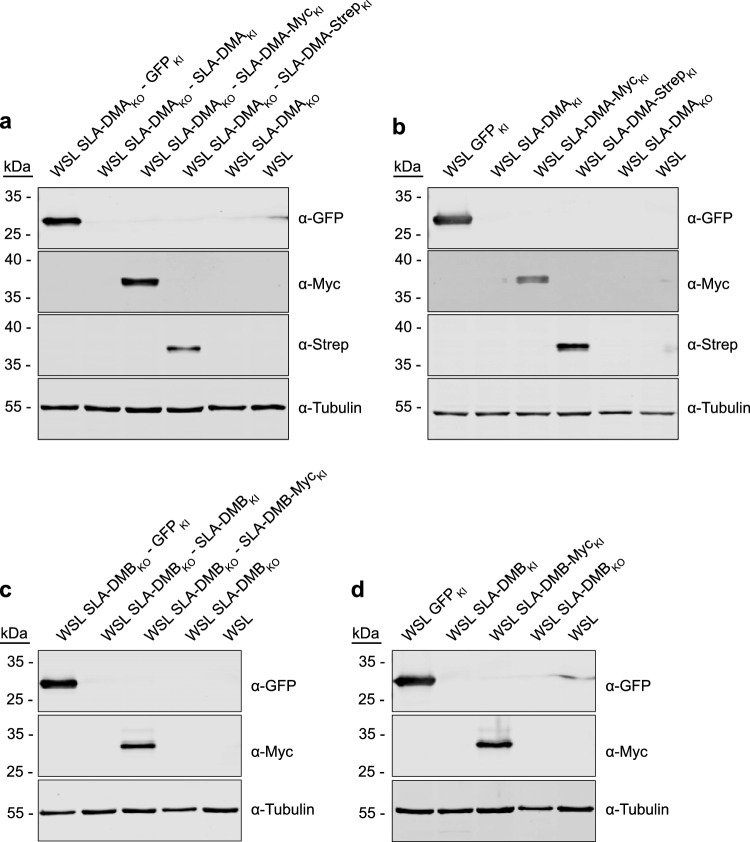
WSL knockout/knockin cells express MHC II transgenes. Lysates of (**a**) WSL SLA-DMA_KO_ cells and (**b**) WSL cells expressing indicated SLA-DMA transgenes or GFP, or lysates of (**c**) SLA-DMB_KO_ cells and (**d**) WSL cells expressing indicated SLA-DMB transgenes or GFP were separated by SDS-PAGE, transferred to nitrocellulose membranes and probed with antibodies against the indicated proteins or protein tags. Molecular masses of marker proteins (in kDa) are indicated on the left. Original blots are presented in Supplementary Fig. 6a–d.

To test whether the observed inhibition of ASFV replication in SLA-DM knockout cells is indeed due to the absence of the respective proteins, WSL SLA-DMA_KO_ (11) and SLA-DMB_KO_ (9) cells were stably transformed with SLA-DMA or SLA-DMB expression cassettes, respectively. The nucleotide sequences of the open reading frames were codon-optimized and modified in the sgRNA target regions by the introduction of silent nucleotide alterations to exclude inactivation of the transgenes by the still integrated CRISPR/Cas9 machinery of the knockout cells (Supplementary Fig. S5). The synthetic genes were cloned with and without tags (StrepII, Myc) into lentivirus expression vectors resulting in pLV-SLA-DMA, pLV-SLA-DMA-Myc, pLV-SLA-DMA-Strep, pLV-SLA-DMB, and pLV-SLA-DMB-Myc, and a GFP expression construct (pLV-GFP) serving as control. Parental WSL cells were transduced with all generated vectors; SLA-DMA_KO_ (11) cells were transduced either with pLV-SLA-DMA, pLV-SLA-DMA-Myc, pLV-SLA-DMA-Strep or pLV-GFP, and SLA-DMB_KO_ (9) cells with pLV-SLA-DMB, pLV-SLA-DMB-Myc or pLV-GFP and stably transformed cells were selected using puromycin containing medium. Unlike in parental WSL and knockout cells, tagged proteins of the expected sizes were detectable by western blotting in the WSL knockout/knockin (WSL_KO/KI_) cells (Fig. 6). Unfortunately, the untagged SLA-DMA and -DMB proteins were not recognized by the available antibodies raised against human leucocyte antigen (HLA)-DMA and HLA-DMB. Nevertheless, these cell lines were included in the analysis of ASFV infection, since the C-terminally added tags might impair maturation, or heterodimeric complex formation between the transgene-encoded and the unaffected endogenous alpha or beta chains of SLA-DM.

**Figure 7:**
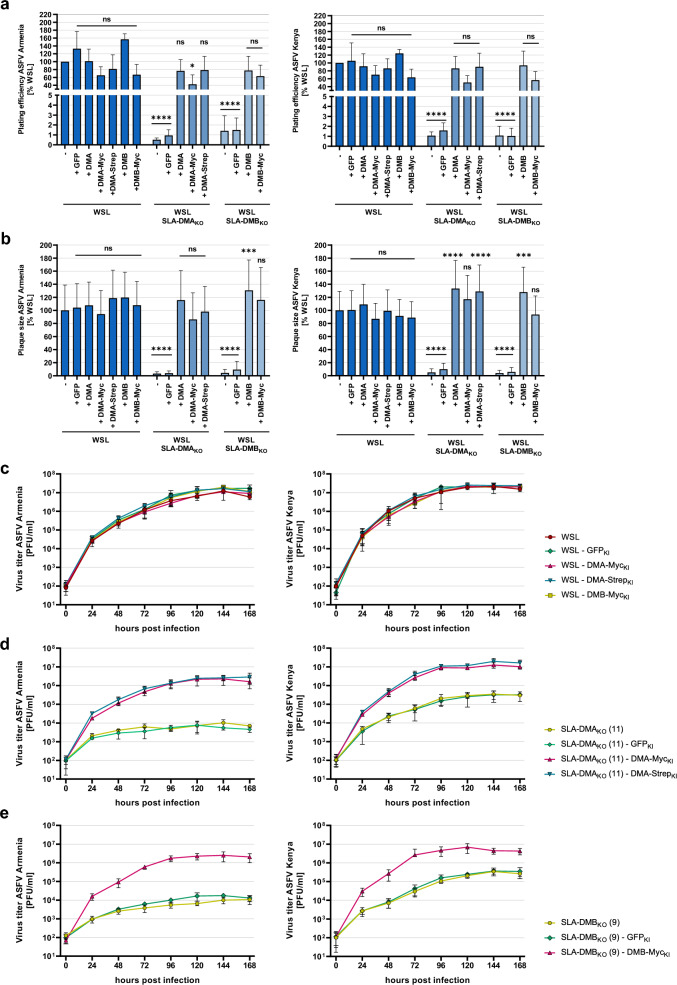
MHC II transgene expression in WSL knockout/knockin cells restored ASFV replication. (**a**, **b**) For the determination of plating efficiency and plaque size ASFV Armenia or ASFV Kenya-infected WSL, WSL_KO_ and WSL_KO/KI_ cells were visualized by immunofluorescence staining. (**a**) Plating efficiency of ASFV Armenia and ASFV Kenya was calculated by counting ASFV-infected cells or plaques in three independent experiments (n = 6). Shown are the mean relative (%) titers compared to those on WSL cells, and standard deviations. Significant differences were calculated by ordinary one-way ANOVA followed by Tukey’s multiple comparison test. * = p < 0.05, **** = p < 0.0001, ns = not significant. (**b**) For the determination of plaque sizes, areas of fifty plaques per cell line from three independent experiments (n = 150) were measured and the mean relative (%) sizes compared to WSL cells including standard deviations are shown. Significant differences were calculated by Kruskal–Wallis test followed by Dunn’s multiple comparison test. *** = p < 0.001, **** = p < 0.0001, ns = not significant. (**c**–**e**) Multi step (MOI 0.02) growth analysis of ASFV Armenia and Kenya in untreated and transgene-expressing lentivirus-transduced (**c**) WSL, (**d**) WSL-DMA_KO_, and (**e**) WSL-DMB_KO_ cells. Shown are the mean results of two independent experiments with two replicates (n = 4) and standard deviations.

In transduced SLA-DMA_KO_ (11) cells plating efficiency of ASFV Armenia and Kenya increased to about 77% and 86%, respectively, of the titers in parental WSL cells after reintroduction of authentic SLA-DMA (SLA-DMA_KO_-DMA_KI_) (Fig. 7a). In SLA-DMA_KO_-DMA-Myc_KI_ cells 42% (Armenia) and 50% (Kenya), and in SLA-DMA_KO_-DMA-Strep_KI_ cells 79% (Armenia) and 90% (Kenya) of the original titers were achieved. In WSL SLA-DMB_KO_-DMB_KI_ cells plating efficiencies of 77% (Armenia) and 94% (Kenya), and in SLA-DMB_KO_-DMB-Myc_KI_ cells plating efficiencies of 63% (Armenia) and 56% (Kenya) were observed (Fig. 7a). In most cases, the apparent virus titers on different WSL_KO/KI_ cells were not significantly lower than on parental WSL cells (Fig. 7a). Thus, the added tags obviously did not (StrepII) or only marginally affect (Myc) the proposed function of SLA-DM for ASFV replication. Additional statistical analyses of the differences observed between the plating efficiencies on knockout, knockin and knockout/knockin cell clones are shown in Supplementary Information.

Stable transduction of WSL SLA-DMA_KO_ and SLA-DMB_KO_ cells with expression cassettes for the corresponding tagged or untagged proteins also restored cell-to-cell spread of ASFV Armenia and ASFV Kenya. In all cases, plaques on different WSL_KO/KI_ cells exhibited similar or even larger sizes than detected on parental WSL cells (Fig. 7b, Supplementary Fig. S6). Statistical analyses of plaque size differences between the individual cell clones are shown in Supplementary Tables S10 and S11.

In line with this, growth kinetic studies revealed that on WSL_KO/KI_ cells the progeny virus titers of ASFV Armenia and Kenya were increased by approx. 2.5 and 1.5 log, respectively, when compared to those on the parental cells (Fig. 7c–e), and final virus titers were again similar to those on original WSL cells. Taken together these results show that the deleterious effects of SLA-DMA/B knockout on susceptibility to ASFV infection can be fully reverted by the expression of corresponding transgenes, demonstrating a crucial specific role of SLA-DM in ASFV replication.

## Discussion

As an intracellular viral pathogen ASFV, in spite of its complex genome and proteome, still requires many host factors for propagation. Using a genome-wide CRISPR/Cas9 knockout screen in a susceptible porcine cell line (WSL) we identified the genes *RFXAP, RFXANK, SLA-DMA, SLA-DMB,* and genes encoding CIITA (*LOC100736732, LOC106509697,* and *LOC100624181*) as top candidates important for ASFV replication. The proteins encoded by these genes are part of the MHC II (SLA II) expression and presentation pathway^58^. Targeted inactivation of RFXAP, CIITA, SLA-DMA and SLA-DMB substantially inhibited replication of genotype II and IX ASFV strains, including the current panzootic virus genotype, which confirmed the importance of certain MHC II molecules for ASFV replication in vitro. Moreover, reconstitution of SLA-DMA or SLA-DMB in corresponding knockout cells fully restored their ASFV replication capacity, and points to a crucial specific role of the MHC II molecule SLA-DM during the viral life cycle.

The MHC II complex is highly conserved in vertebrates and designated as SLA II in swine and HLA II in humans. Pigs possess four MHC II molecules, SLA-DR, SLA-DQ, SLA-DO and SLA-DM^59^. SLA-DR and -DQ are classical MHC II transmembrane cell-surface glycoproteins that present exogenous peptides to the antigen receptor of CD4 + helper T cells, whereas SLA-DM and -DO are termed non-classical MHC II molecules. In humans, they are involved in regulation of antigenic peptide loading onto the classical MHC class II molecules. All MHC II proteins are heterodimers that are composed of α and β chains, and in classical MHC II proteins the a1 and b1 domains form the peptide-binding groove. For the human synthesis and presentation pathway it has been described^60^ that the classical MHC II α and β subunits are synthesized in the ER, where they assemble with the specific chaperone CD74/invariant chain (li). The li promotes MHC II folding, prevents premature peptide binding and sorts the MHC II molecules to late endosomal compartments either directly from the *trans*-Golgi network or by reinternalization from the cell surface. The acidic environment of the endosomes mostly degrades li, but leaves a specific li fragment, the class II invariant chain associated peptide (CLIP), within the MHC II binding groove. The release of CLIP is induced by the non-classical MHC II molecule DM through the promotion of a conformational change, whereby classical MHC II proteins are loaded with higher affinity antigenic peptides. Those peptides result from antigens that were internalized by clathrin-mediated endocytosis, phagocytosis, or micropinocytosis.

ASFV enters its host cells via clathrin-mediated endocytosis or macropinocytosis. Like MHC II DM that is targeted towards late endosomes via a tyrosine motif^60^, ASFV is also trafficking to late endosomes during entry^24–28^. Late endosomes thus represent a cell compartment where MHC II DM (SLA-DM) molecules and incoming ASFV particles come into close proximity. Recently it has been discovered that the endosomal proteins NCP1 and LAMP-1/-2 interact with the putative ASFV fusion proteins pE258R or pE199L, respectively. Moreover, CRISPR/Cas9 induced knockout of NCP1 led to a retention of ASFV particles within late endosomes. Furthermore, a reduction of ASFV infected cells and a decrease in viral replication was observed. As ASFV infection was only partially inhibited it was hypothesized that the virus might be able to utilize alternative endosomal host factors^42^. In our study, SLA-DMA and SLA-DMB knockout cells displayed a severe defect in susceptibility to ASFV infection and subsequent DNA and virus replication. This effect could be reversed by the reintroduction of the respective SLA-DM subunits, indicating that SLA-DM might be important for ASFV entry, e.g. for efficient uncoating and the release of core particles from the late endosomes. Regrettably first attempts to localize ASFV in inoculated SLA-DMA_KO_ and CIITA_KO_ cells by electron microscopy failed, and no accumulations of stalled particles within the late endosomal compartments could be observed, possibly due to their rapid degradation and moderate MOI of 5 wild-type WSL infectious units per cell. However, the absence of viral factories and detectable amounts of progeny virus particles in electron microscopy analyses of WSL_KO_ cells, as well as the substantially reduced plating efficiencies and genome copy numbers of ASFV in WSL_KO_ versus parental wildtype cells strongly indicates that infection of the MHC II-deleted cells is blocked very early.

In the genome-wide CRISPR/Cas9 knockout screens of WSL cells resistant to ASFV infection, sgRNAs targeting *RFXAP, RFXANK, LOC100736732, LOC106509697,* and *LOC100624181* were also significantly elevated. These genes code for RFXAP, RFXANK, and CIITA (*LOC100736732, LOC106509697, LOC100624181*). Together with RFX5 these molecules are involved in the expression of all MHC II genes^61–65^. Our studies revealed that in CIITA_KO_ or RFXAP_KO_ cells, ASFV infection was inhibited to a similar extent as in knockout cells lacking SLA-DMA or SLA-DMB. Unlike in SLA-DM_KO_ cells, MHC II expression was generally inhibited in cells lacking functional CIITA or RFXAP genes. This was shown by IF and MS analyses which did not detect the classical MHC II molecules SLA-DR and SLA-DQ, although they are present in parental WSL cells. Since CIITA and RFXAP are general regulators of MHC II expression^58^ it is likely that corresponding knockout cells also lack the non-classical MHC II molecules SLA-DO and SLA-DM which could not be detected or quantified with our available tools. Although it cannot be excluded that the regulatory proteins CIITA or RFXAP, or SLA-DR, SLA-DQ and SLA-DO are also directly involved in ASFV infection or replication, it appears more likely that SLA-DM is the only relevant MHC II molecule. This is underpinned by the fact that no sgRNA hits targeting SLA-DR, SLA-DQ or SLA-DO were identified in the performed CRISPR/Cas9 knockout screen. Moreover, flow cytometric analysis of susceptible cells indicated that expression of the classical MHC II molecule SLA-DQ is not essential for ASFV infection^66^. In contrast, a CRISPR/Cas9 knockout screen to discover relevant host factors for bat influenza A virus identified besides the MHC II transcription factors RFXANK, RFXAP, RFX5 and CIITA also the classical MHC II molecules HLA-DRA and HLA-DRB1 and downstream experiments confirmed that the MHC II molecule HLA-DR is an essential entry determinant for bat influenza A viruses^46^.

Previous studies revealed that ASFV infection of macrophages downregulates SLA-DMA and SLA-DMB expression, while the expression of SLA-DOA/B is upregulated^67^. The present results indicate that besides the proposed role in immune evasion, this ASFV-induced downregulation of SLA-DM might also prevent reinfection of host cells and, thereby, enhance the efficiency of virus propagation. Modulation of the MHC II-mediated cellular immune response has been also described for human cytomegalovirus infection, where the viral protein pUS2 destroys HLA-DMA and -DRA to prevent recognition by CD4 + T cells^68^.

Expression of MHC II molecules is found predominantly in professional antigen presenting cells (APCs) such as macrophages, B cells and dendritic cells, as well as thymic epithelial cells^69–71^. Additionally, MHC II expression in various capillary endothelia of pigs has been described^72^. At first it appeared surprising that molecules of the MHC II pathway were identified during our CRISPR/Cas9 knockout screens in WSL cells, which are a permanent line derived from wild boar lung. However, previous characterization of WSL cells already showed that these cells express proteins that are specific for the monocyte/macrophage lineage, including SWC3, CD14, CD169, CD163 and SLA II. Therefore, it has been speculated that WSL cells are derived from the myeloid lineage and might represent immature precursors of macrophages^54^. Monocytes and macrophages, which belong to the group of APCs, are the main target cells of ASFV in its vertebrate host^73,74^. Therefore, it should be attempted in future studies to knockdown SLA-DM expression in macrophages e.g. with siRNAs to verify whether this affects ASFV replication also in its natural host cell.

Loss-of function CRISPR/Cas9 screenings are powerful tools to identify host factors required for virus entry and for downstream steps of virus replication. Previous studies of this kind identified cell proteins that are crucial for replication of influenza viruses, flaviviruses, noroviruses, lentiviruses, herpesviruses, and others^44–52^. With the data presented here we could unequivocally demonstrate for the first time that components of the MHC II system, in particular the non-classical membrane protein SLA-DM, are crucial for ASFV replication, at least in cell culture. Further research is required to unravel in detail how ASFV utilizes this molecule of the cellular immune response for its own benefit. Moreover, it has to be investigated whether SLA-DM might be a suitable target protein for antiviral treatment, or development of ASFV-resistant swine.

## Materials and methods

### Cell lines and viruses

Cell lines were received from the cell culture collection for veterinary medicine (CCVM) of the Friedrich-Loeffler-Institut (FLI). The highly passaged wild boar lung cell line (WSL-R-HP, #1346; abbreviated as WSL) was maintained in Ham’s F12 cell culture medium (Ham’s F-12, 5.32 g/L; IMDM, 8.80 g/L; NaHCO_3_, 2.45 g/L; pH 7.2) which was supplemented with 10% fetal calf serum (FCS). Cloning of the cell line was performed by limiting dilution in 96 well plates. Single cells were propagated, and one WSL cell clone with parental phenotype was selected and used for all targeted knockout experiments. A rabbit kidney cell line (RK-13, #0237) was maintained in Minimum Essential Medium (MEM; MEM-Eagle-Hank´s salt, 5.32 g/L; MEM Earle´s salt, 4.76 g/L; NaHCO_3_, 1.25 g/L; non-essential amino acids, 1%; Na-Pyruvate, 0.12 g/L; pH 7.2) supplemented with 10% FCS. The human embryo kidney cell line (HEK293Td4.1, #1539) was also maintained in MEM supplemented with 10% FCS. All cells were incubated at 37 °C and 2.5% CO_2_.

ASFV Armenia 2008 (ASFV Armenia), a virulent genotype II ASFV isolate from Armenia^55^, was kindly provided by Sandra Blome (FLI). The virus was adapted to efficient growth in cell culture through 21 serial passages on WSL cells. The genotype IX isolate ASFV Kenya 1033^55,75^ was kindly provided by Richard Bishop (International Livestock Research Institute, Nairobi, Kenya). The mutant ASFV Kenya 1033 ΔCD2v dsRed containing a reporter gene expression cassette at the deleted CD2v (EP402R) gene locus^55,56^ was kindly provided by Günther M. Keil (FLI). The plasmid-based PrV mutant PrV-BaΔgGG^76^ was used as heterologous control virus.

### Porcine CRISPR library

The generation and characterization of the porcine CRISPR knockout library (SsCRISPRko.v1) has been described^52^. Briefly, for the generation of specific single guide RNAs (sgRNA) targeting protein coding genes the genome assembly *S.* *scrofa* 10.2^77^ was used. Three to four sgRNAs for each gene were selected. In total, the porcine CRISPR library consisted of 83,381 specific sgRNA targeting 20,598 porcine genes and 1001 non-targeting controls, cloned in the pLenti-CRISPRv2 backbone (Addgene #52961).

### Genome-wide CRISPR/Cas9 knockout screen

The genome-wide CRISPR/Cas9 knockout screen was performed as described previously^52^ with slight modifications as described below.

In five 20 cm dishes 5 × 10^6^ WSL cells each were seeded one day before they were transduced with the lentiviral sgRNA library, that was produced in accordance with the protocol by Joung, et al.^78^, at a MOT of 0.3 in medium containing 10 µg/ml polybrene. Three days after transduction, cells were split into eight dishes at a density of 5 × 10^6^ cells/plate with medium containing 1.25 µg/ml puromycin. At confluency, selected transduced cells were split again into a total of 16 plates at a density of 5 × 10^6^ cells/plate. At 12 days post transduction cells were seeded into 30 dishes at a density of 1 × 10^7^ cells/dish for infection the next day. At this time at least 6 × 10^7^ cells were harvested, sedimented and stored as controls at − 20 °C for DNA extraction. Infection was performed with ASFV Kenya 1033 ΔCD2v dsRed at a MOI of 0.3 or 0.5 (depending on the available amounts of virus stocks), since this MOI had been shown to be required to kill at least most of infected WSL control cells. The cells were checked daily for fluorescent marker expression and cytopathic effect, and medium was added or changed as appropriate. For medium changes 20% conditioned medium from untreated WSL cells was included. After approx. four to five weeks growing cell colonies from all plates were trypsinized, and divided into two pooled subsets. At least 2 × 10^7^ cells of each subset were stored at − 20 °C for DNA preparation. The remaining cells were reseeded into cell culture dishes and infected as above. Cells surviving the second infection were harvested approx. 20 days later. Cell collection for DNA preparation, reseeding and infection was repeated four times in total. The entire screening procedure was conducted twice resulting in two sets of uninfected control cells, and eight survivor populations (two sets per screen).

For reliable virus inactivation the sedimented cells were resuspended in TEN (20 mM Tris–HCl, pH 7.4, 1 mM EDTA, 150 mM NaCl) supplemented with RNase A (500 µg/mL, Serva) and incubated for 1 h at 37 °C. After addition of SDS to a final concentration of 0.3%, samples were incubated further at 75 °C for 30 min, before the standard lysis and DNA extraction protocol was executed using sarkosyl buffer, RNase A, pronase, phenol–chloroform extraction, and ethanol precipitation as described before^52^.

To generate sequencing libraries three sequential PCR amplifications of the extracted DNAs were performed. First, four 50 µl reactions per sample containing 5 µg of DNA, 25 fmol each of P5-forward primer (ITA2fwd_P5) and P7-reverse primer (ITA2rev_P7_leCRV) (Supplementary Table S1), and 3.75 U ExTaq DNA polymerase (Clontech) were prepared according to manufacturer’s specifications. Incubation conditions were as follows: 95 °C for 1 min, 28 cycles of 95 °C for 30 s, 53 °C for 30 s, 72 °C for 1 min, 72 °C for 10 min. After amplification, the four PCR reactions of each sample were pooled and the 155 bp products were gel purified using a gel extraction kit (Zymo Research). The DNA was eluted in nuclease-free water and 200 ng DNA were used in a second PCR reaction with the same ingredients as before but with 25 fmol each of a sample-specific P5-barcode-forward primer (e.g. ITA2fwd_ID85_P5both) and P7-reverse primer (e.g. ITA2rev_IDxx_P7leCrv2) (Supplementary Table S1). Reaction conditions were as follows: 95 °C for 1 min, 14 cycles of 95 °C for 30 s, 57 °C for 30 s, 72 °C for 1 min, 72 °C for 10 min. PCR products were purified with the QIAquick Nucleotide Removal Kit (QIAgen), and eluted in 35 µl nuclease-free water. A third amplification step with the entire eluted DNA was conducted in a volume of 300 µl (6 × 50 µl) using the same compounds as for the second PCR except a tenfold higher concentration of the forward and reverse primers (250 fmol). The PCR reaction was performed as before, but only for one single cycle. The 222 bp amplification products were gel purified and eluted in nuclease-free water. The isolation of DNA and the three consecutive PCR amplifications were performed in parallel for the complete sample sets including the corresponding controls to minimize bias.

Samples were sequenced in an IonTorrent Ion S5™ XL System (Invitrogen, Thermo Fisher Scientific) and sequencing data were processed and analyzed on the Galaxy web platform (usegalaxy.eu) using Cutadapt (Galaxy Version 1.16.5), MAGeCK count tool (Galaxy version 0.5.8.4), and MAGeCK test tool (Galaxy version 0.5.8.1) as described^52,57,79,80^. The sequencing results of two different conditions (‘Control’ vs. ‘Survivor’) were compared using the robust rank aggregation (RRA) method of the MAGeCK test tool^57^.

### Generation of WSL knockout cells

For the targeted generation of SLA-DMA, SLA-DMB, CIITA, or RFXAP knockout cells one of the four gene specific sgRNAs from the whole genome library was selected (Supplementary Fig. S1 and Table S4) and cloned into the multiplex CRISPR/Cas9 vector pX330A-1×4 which was a gift from Takashi Yamamoto (Addgene plasmid # 58768; http://n2t.net/addgene:58768; RRID:Addgene_58768)^81^ and which was modified to express a neomycin resistance gene (neoR; designated as pX330A-1×4neoRA). To obtain pX330A-1×4neoRA the 8962 bp vector pX330A-1×4 was linearized within a non-functional region by digestion with PciI, and neoR under control of the simian virus 40 (SV40) early promoter was inserted as a 1667 bp PciI fragment isolated from pX330-ΔNLS1/2neoR^55^. In the resulting plasmid this resistance gene was in parallel orientation to the sgRNA and Cas9 genes. Next, complementary DNA oligonucleotides containing the target-specific sequences of the sgRNAs with matching 5’ overhangs (Eurogentec; Supplementary Table S7) were hybridized, phosphorylated, and cloned into BpiI-digested and dephosphorylated pX330A-1×4neoRA. Accuracy of resulting plasmids was checked by sequencing with the HU6-SF primer (Supplementary Table S5). Plasmids pX330A-1×4neoRA-SLA-DMA gR2, -SLA-DMB gR3, -MHCIITA gR2, and -RFXAP gR2, were transfected into cloned WSL cells using the K2® Transfection System (Biontex) according to manufacturer’s instructions. After three days the cells were trypsinized, serially diluted and seeded into 96 well plates using media supplemented with 0.5 mg/ml G418 sulfate (Invitrogen, Thermo Fisher Scientific). Resistant single cell clones were further propagated and checked by immunoblot for Cas9 expression using an anti-FLAG antibody (see below). DNA of Cas9 positive cells was prepared using the QIAamp DNA Mini Kit (QIAgen) according to manufacturer’s instructions, and used for PCR and subsequent sequencing of the PCR products to confirm the integration of the sgRNA sequences as well as insertions or deletions of nucleotides within the targeted genes. Primers used for PCR and sequencing are listed in Supplementary Table S5.

### Generation of SLA-DM reconstituted cell lines

Coding sequences of SLA-DMA (GenBank #NC_010449.5, nt 25133494 to 25137928) and SLA-DMB (GenBank #NC_010449.5, nt 25119278 to 25125089) were spliced in silico and codon optimized. It was also ensured that the binding regions of the selected sgRNA were altered as far as possible by silent base substitutions (Supplementary Fig. S5). The custom-made plasmids (Invitrogen, Thermo Fisher Scientific) pMA-SLA-DMA and -DMB contained 5’-EcoRI and 3’-NotI restriction sites for convenient recloning of the ORFs, and unique BpiI cleavage sites immediately upstream of the termination codons, which permitted in-frame insertion of hybridized oligonucleotides (Eurofins Genomics; Supplementary Table S7), encoding a StepII-tag (WSHPQFEK) or a Myc-tag (LEQKLISEEDL), respectively, into the BpiI- and NotI-digested constructs. Correct insertions were verified by sequencing using primer M13 Rev (-24) (Supplementary Table S5). The native and StrepII- or Myc-tagged SLA-DMA ORFs, as well as the native and Myc-tagged SLA-DMB ORFs were recloned as EcoRI/NotI fragments into the correspondingly digested 8140 bp lentivirus vector pLVX-IRES-Puro (TaKaRA/Clontech). As a control the ORF encoding enhanced green fluorescent protein (EGFP) isolated as a 772 bp EcoRI/NotI fragment of pEGFP-N1 (Clontech) was also inserted, resulting in pLVX-EGFP-IRES-Puro. Correct plasmid clones were identified by sequencing using primer CMV promotor-F (Supplementary Table S5). Protein expression was confirmed in RK13 cells transfected (X-tremeGENE™ HP reagent, Roche) with the newly generated plasmids and immunoblot analysis using the anti-Myc and anti-Strep antibodies.

Lentiviruses encoding the SLA-DMA-Strep, SLA-DMA-Myc, SLA-DMB-Myc and EGFP gene, respectively, or empty pLVX-IRES-Puro were generated in HEK-293 T cells in accordance with the protocol described by Joung, et al.^78^ WSL, WSL SLA-DMA_KO_ (11), and WSL SLA-DMB_KO_ (9) cell clones were transduced and knockout/knockin (WSL_KO/KI_) cells were selected using 1 µg/ml puromycin. Knockout of the native gene and transgene integration was confirmed by PCR and sequencing using primers listed in Supplementary Table S5. Transgene expression was again confirmed by immunoblotting as described below.

### Sanger sequence analyses

The generated plasmids and PCR-amplified (KOD Xtreme Hot Start DNA Polymerase, Merck) relevant genome fragments of recombinant WSL cell lines were sequenced with the indicated primers and the BigDye™ Terminator v1.1 Cycle Sequencing Kit (Thermo Fisher Scientific), in an Applied Biosystems 3500 Genetic Analyzer (Thermo Fisher Scientific). Results were evaluated using the Geneious Prime 2021.0.1 software package (Biomatters, available from https://www.geneious.com).

### Immunoblotting

Cells were trypsinized, resuspended in medium containing 10% FCS, centrifuged, and washed once with phosphate buffered saline (PBS). The sedimented cells were then lysed in sodium dodecyl sulfate–polyacrylamide (SDS) containing sample buffer (0.13 M Tris–HCl, pH 6.8; 4% SDS; 20% glycerol; 0.01% bromophenol blue; 10% 2-mercaptoethanol), sonicated and incubated for 5 min at 95 °C. Proteins were separated in discontinuous SDS polyacrylamide gels and transferred to nitrocellulose membranes. Blots were blocked for 3 h at RT with 5% skim milk in tris buffered saline with 0.25% Tween 20 (TBS-T), and probed overnight with specific primary antibodies diluted in 0.5% skim milk in TBS-T. Binding of monoclonal anti-FLAG (clone M2, #F1804, Sigma-Aldrich), anti α-tubulin (#T5168, Sigma-Aldrich), and polyclonal rabbit anti-StrepII (#4217, ProSci), anti-Myc (#PA1-581, Invitrogen, Thermo Fisher Scientific), anti-HLA-DMA (H00003108-D01P, Abnova), and anti-HLA-DMB (H00003109-D01P, Abnova) antibodies was visualized with secondary fluorophore-labelled donkey anti-rabbit IRDye 800CW (#926-32213, Li-Cor Biosciences) or donkey anti-mouse IRDye 680RD (#926-68072, Li-Cor Biosciences) antibodies in TBS-T. Fluorescent signals were detected with an Odyssey CLx infrared imaging system (CLX-2293; Li-Cor Biosciences).

### Determination of plaque size and plating efficiency

WSL, WSL_KO_ and WSL_KO/KI_ cells were seeded at a density of 4 × 10^5^ cells per well in 24 well plates. The next day viruses were serially diluted in cell culture medium supplemented with 5% FCS and applied to the confluent cell layers. The cells were incubated for 2 h at 37 °C and 2.5% CO_2_. Subsequently, the inoculum was removed and replaced with methocel medium (6 g/L methyl cellulose in MEM with 5% FCS). PrV infection was visualized and documented as described below by the inherent GFP expression of PrV-BaΔgGG three days after infection. Four days after ASFV infection the medium was removed and cells were washed once with PBS before they were fixed with 4% paraformaldehyde in PBS (PFA) for 20 min at room temperature (RT). Formaldehyde fixation was stopped by washing and subsequent incubation with 5 mM NH_4_Cl in PBS for 30 min at RT. Cells were washed three times with PBS and stored at 4 °C until ASFV antigen detection by indirect immunofluorescence (IF) tests (see below).

ASFV and PrV-infected cells, foci and plaques were visualized with a Leica DMi8 motorized fluorescence microscope. Using the Leica Application Suite X software whole wells were imaged and resulting mosaic images were merged. For each virus and each cell line the areas of 50 infected cells or plaques were determined using the freehand selection tool of ImageJ (Version 1.53f51; http://imagej.nih.gov/ij) in three independent experiments. The mean plaque sizes of respective viruses grown on WSL cells were set to 100% and mean relative plaque sizes of viruses grown on WSL_KO_ or WSL_KO/KI_ cells as well as standard deviations were calculated using GraphPad Prism (Version 9). For plating efficiency plaques were counted and apparent titers were calculated as PFU/ml.

### Determination of viral titers and replication kinetics

WSL, WSL_KO_, and WSL_KO/KI_ cells were seeded at a density of 4 × 10^5^ cells (for PrV infection) or 3 × 10^5^ cells (for ASFV infection) per well in 24 well plates. One day later PrV-BaΔgGG, ASFV Armenia, or ASFV Kenya, were applied at a MOI of 0.02. After an incubation at RT (PrV) or 37 °C (ASFV) for 2 h, cells were washed once with medium and subsequently overlaid with 1 ml medium containing 1% penicillin/streptomycin (Gibco). PrV-infected cells were frozen three days after infection at  -80 °C. Single plates of ASFV-infected cells were frozen immediately after the addition of medium, as well as every 24 h until 168 h p.i.. For titration, plates were thawed and lysates were transferred to reaction tubes. After centrifugation at 2655 xg and 4 °C for 5 min supernatants were transferred into fresh tubes and stored at − 80 °C. Virus titrations were performed on confluent RK13 cells (PrV) or WSL cells (ASFV) in 96 well plates. After inoculation with serial dilutions of the virus supernatants (100 µl/well), cells were incubated for 2 h at RT on a rocker (PrV), or centrifuged for 1 h at 689 xg and 37 °C (ASFV) before the virus supernatant was removed and cells overlaid with methocel medium. The cells were incubated for 3 days (PrV) or 4 days (ASFV) at 37 °C in a 2.5% CO_2_ atmosphere. PrV-infected cells were fixed for 1 h by addition of a 3.7% formaldehyde solution, and subsequently stained with 1% crystal violet to visualize the plaques. Cells infected with ASFV were washed once with PBS, fixed with ice cold acetone/methanol (1:1, v/v) for 30 min at − 20 °C, and air dried. Infected cells were visualized by IF staining.

### DNA replication kinetics

For the analysis of viral DNA replication WSL and WSL_KO_ cells were seeded at a density of 3 × 10^5^ cells in 24 well plates and infected 24 h later with ASFV Armenia at a MOI of 3 in two replicas for each time. After an incubation of 2 h at 37 °C the inoculum was removed, and after a wash, replaced with medium. After 0, 2, 4, 8, 16 and 32 h at 37 °C, the medium was aspirated again and the cells were washed once with PBS, pelleted and stored until further analysis at -20 °C. DNA was prepared with the NucleoMag Tissue Kit (Macherey–Nagel) according to manufacturer’s recommendations, and eluted in 100 µl elution buffer.

Quantitative real-time PCR for DNA detection was performed using the QuantiTect Multiplex PCR NoROX kit (Qiagen) in 12.5 µl reactions containing 2.5 µl of infected cell DNA according to the manufacturer’s instructions. The ASFV B646L gene-specific primer pairs AKB646L-408F and AKB646L-507R, as well as the β-actin gene-specific primer pair ACT-CP-F and ACT-CP-R (Supplementary Table S5) were included at 800 nM, and the TaqMan probes AKB646L-460P and ACT-CP-P (Supplementary Table S5) at 160 nM final concentrations. Primer and probes were purchased from Eurogentec. Samples were incubated for 15 min at 95 °C followed by 45 cycles of 30 s 95 °C, 30 s 55 °C, and 30 s 68 °C in a Bio-Rad C1000/CFX96 real-time PCR machine, and results were analyzed using CFX Maestro software (Bio-Rad). ASFV genome copy numbers were determined based on standard curves generated by reactions containing 10^10^, 10^8^, 10^6^, 10^4^, 10^2^ or 10^0^ copies of a p72 expression plasmid (pCAGGS-p72-Georgia, kindly provided by G.M. Keil).

### Indirect immunofluorescence (IF) analysis

After fixation of the cells with PFA as described above, cells were optionally permeabilized with 0.5% Triton-X 100 in PBS for 15 min at RT. This step was not required for the visualization of surface proteins, or after fixation with acetone/methanol. After washing with PBS, the cells were blocked with 10% FCS in PBS for 1 h at RT, the polyclonal rabbit anti-ASFV p72 antibody^82^ or the monoclonal mouse anti-pig MHC II (clone MSA 3) antibody (kindly provided by Luise Hartmann and Ulrike Blohm) diluted in blocking buffer were applied for 1 h at RT, and detected with goat anti-rabbit Alexa-Fluor 488 (#A11008, Invitrogen, Thermo Fisher Scientific), or goat anti-mouse Alexa-Fluor 488 (#A11001, Invitrogen, Thermo Fisher Scientific) secondary antibodies diluted in PBS for another hour. For the detection of p72 antigen in WSL_KO/KI_ cells, the secondary antibody goat anti rabbit Alexa-Fluor 647 (#A21245; Invitrogen, Thermo Fisher Scientific) was used to enable visualization also in the GFP expressing control cell lines. Nucleic acids were stained with Hoechst 33342 (#H3570; Invitrogen, Thermo Fisher Scientific) for 15 min at RT. After each incubation step the cells were washed three times with PBS, and finally analyzed with a Leica DMi8 fluorescence microscope.

### Mass spectrometry

Confluent monolayers of WSL, WSL DMA_KO_ (11), WSL DMB_KO_ (9), WSL CIITA_KO_ (1) and WSL RFXAP_KO_ (6) (n = 3 per clone) were lysed in 2% SDS in 0.1 M Tris-HCl (pH 8.0) for 10 min at 95 °C. Lysates were clarified by centrifugation (14,000 ×g, 10 min, RT) and the supernatants were collected. Aliquots containing 100 µg protein (determined by BCA assay) were precipitated by addition of 3 volumes of ice-cold acetone. Protein pellets were recovered by centrifugation at 10,000 ×g at 4 °C for 15 min and digested into peptides using the EasyPep™ Mini MS Sample Prep Kit (Thermo Scientific) according to the manufacturers protocol.

Peptides were resuspended in 0.1% formic acid (FA) and peptide yields assessed by BCA assay. Peptides (1 µg/sample) were separated on a nanoElute® (Bruker, Bremen, Germany) HPLC equipped with an IonOpticks Aurora column (25 cm × 75 µm ID, 1.6 µm C18) at a temperature of 40 °C with a flow rate of 400 nL/min coupled to a timsTOF Pro instrument (Bruker). Solvent A was 0.1% FA and solvent B 0.1% FA in acetonitrile. Peptides were eluted with a gradient from 2 to 15% solvent B (0–60 min), 15–24% solvent B (60–90 min), 24%-34% solvent B (90–105 min), 34–95% solvent B (105–107 min). The timsTOF Pro instrument was equipped with a CaptiveSpray nano electrospray ion source (Bruker) and was operated in Parallel Accumulation and Serial Fragmentation (PASEF) mode using the standard DDA method for proteome analysis (1.1 s cycle time) recommended by the manufacturer.

Raw MS-data were processed with Fragpipe^83^ using a data base with porcine sequences downloaded from Ensembl repository^84^. Qualitative and quantitative analysis of protein identifications was performed using the statistical language R^85^ and Perseus v1.6.15.0^86^. The R-package gprofiler2 version 0.2.1^87^ was used to reference porcine protein identifiers to the corresponding genes (HGNC nomenclature).

### Electron microscopy

WSL, WSL SLA-DMA_KO_ and WSL CIITA_KO_ cells were seeded in 6 well plates at a density of 1.5 × 10^6^ cells/well. 24 h later one complete plate of each type was infected with ASFV Armenia at a MOI of 5. The virus was allowed to penetrate the cells for 2 h at 37 °C. Afterwards the virus suspension was removed and replaced with fresh medium containing 1% penicillin/streptomycin. 16 h after application of the virus, cells were scraped into the medium and transferred into a 50 ml centrifuge tube. The cell suspension was centrifuged for 7 min at 350 × g at 4 °C. Cells were washed once with 0.1 M sodium cacodylate buffer (pH 7.2), before they were fixed with 2.5% glutaraldehyde in cacodylate buffer (both SERV Electrophoresis) for at least 2 h at 4 °C. Fixed cells were centrifuged again (5 min, 1000 × g, 4 °C) and the pellet was embedded in low-melting agarose (Sigma Aldrich). After drying the agarose was cut into small pieces (1 mm^3^), post fixed in 1% aqueous OsO_4_ and stained in 2.5% uranyl acetate (both SERVA Electrophoresis). After a stepwise dehydration in ethanol the samples were cleared in propylene oxide and infiltrated with Glycid Ether 100 (SERVA Electrophoresis). For polymerization, samples were filled in capsules and incubated for 3 days at 60 °C. The point of interest was trimmed, and prepared ultrathin sections were transferred to formvar coated nickel grids (Plano, Wetzlar, Germany). All grids were counterstained with uranyl acetate and lead citrate before examination with a Tecnai Spirit transmission electron microscope (FEI, Eindhoven, The Netherlands) at an accelerating voltage of 80 kV.

### Statistical analysis

All statistical analyses were conducted by using GraphPad Prism (Version 9.0). Statistical significance of differences in plating efficiency was calculated by ordinary one-way ANOVA followed by Tukey’s multiple comparison test. Statistical significance of differences in plaque size was estimated by Kruskal–Wallis test followed by Dunn’s multiple comparison test. The number of times the measurements were repeated is indicated in each figure legend. A *p*-value < 0.05 was considered significant and is presented in the figures in form of asterisks (* < 0.05, ** < 0.01, *** < 0.001, **** < 0.0001).

## Supplementary Information

Supplementary Information 1.

Supplementary Information 2.

## Data Availability

Mass spectrometry proteomics data have been deposited to the ProteomeXchange Consortium (http://proteomecentral.proteomexchange.org) via the PRIDE partner repository with the dataset identifier PXD034242. All data needed to evaluate the conclusions in the paper are present in the paper and/or the Supplementary Materials.
