# Supplementary material for: The non-classical major histocompatibility complex II protein SLA-DM is crucial for African swine fever virus replication
Source: Sci Rep. 2023 Aug 21;13:10342. doi: 10.1038/s41598-023-36788-9 (PMC10442341; doi:10.1038/s41598-023-36788-9)
Supplement: Supplementary file 1 — Supplementary Information 1. [file 41598_2023_36788_MOESM1_ESM.pdf]

Supplementary Information

The non-classical major-histocompatibility-complex II protein SLA-DM is crucial for African swine fever virus replication

Katrin Pannhorst[1*], Jolene Carlson[1,#a], Julia E. Hölper[1], Finn Grey[2], John Kenneth Baillie[2], Dirk Höper[3], Elisabeth Wöhnke[1], Kati Franzke[4], Axel Karger[1], Walter Fuchs[1], Thomas C. Mettenleiter[5]

[1] Institute of Molecular Virology and Cell Biology, Friedrich-Loeffler-Institut, Greifswald-Insel Riems, Germany

[2] The Roslin Institute, University of Edinburgh, Midlothian, UK

[3] Institute of Diagnostic Virology, Friedrich-Loeffler-Institut, Greifswald-Insel Riems, Germany

[4] Institute of Infectology, Friedrich-Loeffler-Institut, Greifswald-Insel Riems, Germany

[5] Friedrich-Loeffler-Institut, Greifswald-Insel Riems, Germany

[#a] Current Address: Ceva Animal Health, Greifswald-Insel Riems, Germany

*Correspondence:
Katrin Pannhorst, Friedrich-Loeffler-Institut, Institute of Molecular Virology and Cell Biology, Südufer 10, 17493 Greifswald-Insel Riems, Germany. E-mail address: katrin.pannhorst@fli.de

Table of contents

I. Supplementary tables 3

Table S1. Oligonucleotide sequences for Ion Torrent Sequencing. 3

Table S2. MAGeCKs test gene summary. 4

Table S3. MAGeCKs sgRNA summary. 5

Table S4. Selected sgRNA sequences for the generation of WSL knockout cells........ 11

Table S5. Primers used for PCR, sequencing and qPCR. 12

Table S6. List of identified proteins in WSL and WSL knockout cells. 12

Table S7. Oligonucleotides used for cloning of selected sgRNA sequences into vector pX330A-1x4neoRA as well as for cloning of StrepII- and Myc-tag. 13

Table S8. Statistical analysis of plating efficiency of ASFV Armenia grown on WSL, WSL knockout and WSL knockout / knockin cells........ 14

Table S9. Statistical analysis of plating efficiency of ASFV Kenya grown on WSL, WSL knockout and WSL knockout / knockin cells........ 15

Table S10. Statistical analysis of plaque sizes of ASFV Armenia grown on WSL, WSL knockout and WSL knockout / knockin cells........ 16

Table S11. Statistical analysis of plaque sizes of ASFV Kenya grown on WSL, WSL knockout and WSL knockout / knockin cells. 17

II. Supplementary figures........ 18

Figure S1. Schematic presentation of selected hits found by the genome-wide CRISPR/Cas9 knockout screen........ 18

Figure S2. Characterization of WSL gene knockout cell clones........ 19

Figure S3. Principal component analysis of WSL and WSL knockout proteomes. 20

Figure S4. Growth properties of PrV in WSL knockout cells........ 21

Figure S5. Synthetic open reading frames of SLA-DMA and SLA-DMB. 22

Figure S6. ASFV plaque sizes on WSL, WSL knockout and WSL knockout / knockin cells.... 23

III. Original immunoblots........ 24

Original immunoblot for Fig. 6a 24

Original immunoblot for Fig. 6b 26

Original immunoblot for Fig. 6c 28

Original immunoblot for Fig. 6d 29

IV. Supplementary references 30

I. Supplementary tables

Table S1. Oligonucleotide sequences for Ion Torrent Sequencing.

Primer Name	Direction	Sequence 5' to 3'
ITA2fwd_ID85_P5both	Forward	CCATCTCATCCCTGCGTGTCTCCGACTCAGCCAGCCTCAACGATTTGTGGAAAGGACGAAACACCG
ITA2fwd_ID86_P5both	Forward	CCATCTCATCCCTGCGTGTCTCCGACTCAGCTTGGTTATTCGATTTGTGGAAAGGACGAAACACCG
ITA2fwd_ID87_P5both	Forward	CCATCTCATCCCTGCGTGTCTCCGACTCAGTTGGCTGGACGATTTGTGGAAAGGACGAAACACCG
ITA2fwd_ID88_P5both	Forward	CCATCTCATCCCTGCGTGTCTCCGACTCAGCCGAACACTTCGATTTGTGGAAAGGACGAAACACCG
ITA2fwd_ID89_P5both	Forward	CCATCTCATCCCTGCGTGTCTCCGACTCAGTCCTGAATCTCGATTTGTGGAAAGGACGAAACACCG
ITA2fwd_ID90_P5both	Forward	CCATCTCATCCCTGCGTGTCTCCGACTCAGCTAACCACGGCGATTTGTGGAAAGGACGAAACACCG
ITA2fwd_ID91_P5both	Forward	CCATCTCATCCCTGCGTGTCTCCGACTCAGCGGAAGGATGCGATTTGTGGAAAGGACGAAACACCG
ITA2fwd_ID92_P5both	Forward	CCATCTCATCCCTGCGTGTCTCCGACTCAGCTAGGAACCGCGATTTGTGGAAAGGACGAAACACCG
ITA2fwd_ID93_P5both	Forward	CCATCTCATCCCTGCGTGTCTCCGACTCAGCTTGTCCAATCGATTTGTGGAAAGGACGAAACACCG
ITA2rev_IDxx_P7leCrv2	Reverse	CCTCTCTATGGGCAGTCGGTGATCCAATTCCCACTCCTTTCAAGACCT
ITA2fwd_P5	Forward	TTGTGGAAAGGACGAAACACCG
ITA2rev_P7_leCRV	Reverse	CCAATTCCCACTCCTTTCAAGACCT

Table S2. MAGeCKs test gene summary. Gene summary output of the MAGeCKs test analysis of sequencing data from the performed CRISPR/Cas9 knockout screen 1 (subset 1 and 2) and screen 2 (subset 1 and 2). Shown is data on the positive selection for the best 10 gene hits.

Screen	id	num	pos| score	pos| p-value	pos| fdr	pos| rank	pos| goodsgrna	pos| lfc
**Screen 1 Subset 1**	LOC100736732	4	2.99E-14	0.5	1	1	4	8.5018
	SLA-DMB	4	4.64E-14	0.5	1	2	4	8.5693
	SLA-DMA	4	1.48E-09	1	1	3	3	6.9538
	LOC102165390	4	2.22E-09	1	1	4	4	3.2266
	LOC106509697	4	8.23E-09	1	1	5	3	6.3623
	RFXAP	4	3.01E-07	1	1	6	3	4.8524
	RFXANK	4	4.45E-07	1	1	7	3	4.441
	LOC100624181	4	1.65E-06	1	1	8	2	4.649
	CCZ1	4	4.98E-05	1	1	9	2	3.0009
	MARCO	4	5.85E-05	1	1	10	2	-0.69359
**Screen 1 Subset 2**	LOC100736732	4	6.27E-14	0.5	1	1	4	7.5701
	SLA-DMB	4	5.76E-12	0.5	1	2	4	7.7873
	LOC106509697	4	4.56E-11	0.5	1	3	4	5.7558
	RFXANK	4	1.89E-10	0.5	1	4	4	5.5539
	SLA-DMA	4	4.69E-09	1	1	5	3	6.2778
	LOC100624181	4	1.43E-07	1	1	6	3	5.3919
	LOC102165390	4	1.56E-07	1	1	7	3	4.0599
	CCZ1	4	5.67E-07	1	1	8	2	0.95605
	RFXAP	4	1.57E-06	1	1	9	2	-0.25885
	LYPD4	4	5.50E-05	1	1	10	2	0.16788
**Screen 2 Subset 1**	TMEM30A	4	1.92E-13	0.5	1	1	4	8.0873
	SLA-DMB	4	5.31E-11	0.5	1	2	3	9.7031
	LOC100736732	4	8.68E-10	0.5	1	3	3	9.011
	LOC106509697	4	5.26E-08	1	1	4	3	3.6662
	RFXAP	4	9.45E-08	1	1	5	3	5.9151
	LOC100624181	4	4.09E-07	1	1	6	2	1.2169
	SLA-DMA	4	1.91E-06	1	1	7	2	1.6425
	RFXANK	4	7.23E-06	1	1	8	2	-0.82204
	VPS33A	4	1.92E-05	1	1	9	2	-0.88543
	VPS18	4	2.13E-05	1	1	10	2	2.5772
**Screen 2 Subset 2**	SLA-DMB	4	1.11E-13	0.5	1	1	4	8.5649
	LOC100736732	4	3.73E-12	0.5	1	2	4	8.0343
	CCZ1	4	3.77E-10	0.5	1	3	3	8.7348
	RFXAP	4	1.55E-08	1	1	4	3	5.6714
	RFXANK	4	1.70E-08	1	1	5	3	5.421
	TMEM30A	4	3.18E-08	1	1	6	3	4.2994
	VPS33A	4	4.34E-08	1	1	7	3	4.2248
	SLA-DMA	4	1.77E-07	1	1	8	3	2.3592
	LOC106509697	4	2.17E-06	1	1	9	2	3.3197
	LOC100624181	4	7.07E-06	1	1	10	2	-0.53825

Id: gene ID; num: number of targeting sgRNAs for each gene; pos|score: RRA lo value of this gene in positive selection; pos|p-value: raw p-value of this gene in positive selection; pos|fdr: false discovery rate of this gene in positive selection; pos|rank: ranking of this gene in positive selection; pos|goodsgrna: number of "good" sgRNAs: i.e.: sgRNAs whose ranking is below the alpha cutoff (determined by the --gene-test-fdr-threshold option): in positive selection; pos|lfc: log fold change of this gene in positive selection [1].

Table S3. MAGeCKs sgRNA summary. sgRNA summary output of the MAGeCKs test analysis of sequencing data from the performed CRISPR/Cas9 knockout screen 1 (subset 1 and 2) and screen 2 (subset 1 and 2). Shown is data for the best 10 gene hits.

Screen	sgRNA	gene	control _count	treatment _count	control _mean	treat _mean	LFC	control _var	adj _var	score	p.low	p.high	p.twosided	FDR	high_in _treat
**Screen 1**	sgRNA_15503	LOC100736732	95.339	42377/1.3066e+05/42135/1495.1	95.339	42256	8.7769	3.16E+09	82392	146.88	1	0	0	0	True
**Subset 1**	sgRNA_15501	LOC100736732	163.57	38515/1.2968e+05/83340/25907	163.57	60927	8.5322	2.32E+09	2.44E+05	122.95	1	0	0	0	True
	sgRNA_15502	LOC100736732	91.6	27953/92746/37773/1213.1	91.6	32863	8.4713	1.55E+09	76018	118.86	1	0	0	0	True
	sgRNA_15504	LOC100736732	174.79	37981/1.0871e+05/44978/904.02	174.79	41479	7.8824	2.06E+09	2.79E+05	78.18	1	0	0	0	True
	sgRNA_35433	SLA-DMB	0	8378.7/25005/7394.5/169.56	0.9347	7886.6	11.993	1.18E+08	8.3517	2728.7	1	0	0	0	True
	sgRNA_35432	SLA-DMB	158.9	51831/1.6342e+05/93072/7991.8	158.9	72452	8.8237	4.43E+09	2.30E+05	150.61	1	0	0	0	True
	sgRNA_35431	SLA-DMB	80.384	21548/41811/30281/4386.4	80.384	25914	8.3149	2.51E+08	58444	106.86	1	0	0	0	True
	sgRNA_35434	SLA-DMB	196.29	13734/1.087e+05/66345/4108.3	196.29	40039	7.665	2.46E+09	3.53E+05	67.103	1	0	0	0	True
	sgRNA_35437	SLA-DMA	70.102	19782/44494/19839/894.33	70.102	19811	8.1222	3.22E+08	44374	93.711	1	0	0	0	True
	sgRNA_35438	SLA-DMA	35.518	4726.5/21901/7148.9/124.99	35.518	5937.7	7.3454	9.72E+07	11302	55.519	1	0	0	0	True
	sgRNA_35436	SLA-DMA	35.518	4520.9/5715.5/2378.7/31.006	35.518	3449.8	6.5622	6.37E+06	11302	32.116	1	2.06E-226	4.12E-226	5.73E-223	True
	sgRNA_35435	SLA-DMA	8.4123	97.203/3.5314/0/0	8.4123	1.7657	-1.7669	3039.2	627.84	1.7219	0.042546	0.95745	0.085093	0.089344	False
	sgRNA_31072	LOC102165390	156.09	4720.4/5750.8/32.144/0	156.09	2376.3	3.9196	9.34E+06	2.22E+05	4.7091	1	1.98E-06	3.95E-06	0.0012337	True
	sgRNA_31074	LOC102165390	37.388	1179.6/776.9/0/0	37.388	388.45	3.3427	3.60E+05	12530	3.1363	0.99864	0.0013562	0.0027124	0.0048485	True
	sgRNA_31075	LOC102165390	108.42	1736.5/3475.8/151.72/0	108.42	944.1	3.1105	2.85E+06	1.07E+05	2.5579	0.99164	0.0083578	0.016716	0.018829	True
	sgRNA_31073	LOC102165390	200.96	1124.9/4594.3/830.61/0.96894	200.96	977.76	2.2769	4.69E+06	3.70E+05	1.2776	0.84005	0.15995	0.31989	0.32494	True
	sgRNA_15500	LOC106509697	63.559	15547/73017/1.5337e+05/30949	63.559	51983	9.6532	4.16E+09	36434	272.01	1	0	0	0	True
	sgRNA_15499	LOC106509697	128.05	13814/38611/19766/771.27	128.05	16790	7.0236	2.50E+08	1.49E+05	43.136	1	0	0	0	True
	sgRNA_15498	LOC106509697	117.77	3992.4/8362.3/12772/953.43	117.77	6177.3	5.701	2.68E+07	1.26E+05	17.066	1	2.10E-65	4.20E-65	3.30E-62	True
	sgRNA_15497	LOC106509697	71.037	272.37/0/0/0	71.037	0	-6.1707	24728	45573	3.0261	0.0012386	0.99876	0.0024772	0.0048138	False
	sgRNA_48311	RFXAP	98.143	20882/1.3413e+05/64029/1576.5	98.143	42456	8.7423	3.67E+09	87342	143.32	1	0	0	0	True
	sgRNA_48312	RFXAP	172.92	20234/44838/7769.9/118.21	172.92	14002	6.3312	4.07E+08	2.73E+05	26.461	1	2.19E-154	4.38E-154	5.14E-151	True
	sgRNA_48314	RFXAP	205.63	48.601/0/0/0	205.63	0	-7.6909	787.36	3.87E+05	3.3355	0.00042576	0.99957	0.00085152	0.0048138	False
	sgRNA_48313	RFXAP	163.57	153.9/3212.7/4981.1/196.69	163.57	1704.7	3.3736	5.90E+06	2.44E+05	3.1184	0.99856	0.001444	0.002888	0.0049135	True
	sgRNA_82036	RFXANK	49.539	6408.3/10441/1648.4/3.8757	49.539	4028.3	6.317	2.29E+07	22067	26.784	1	3.94E-158	7.88E-158	9.38E-155	True
	sgRNA_82033	RFXANK	139.27	14060/14012/1742.2/0.96894	139.27	7876.9	5.8115	5.85E+07	1.77E+05	18.408	1	8.93E-76	1.79E-75	1.50E-72	True
	sgRNA_82034	RFXANK	93.47	2413.9/1454/131.15/0	93.47	792.6	3.0705	1.38E+06	79172	2.4847	0.98971	0.010289	0.020579	0.022884	True
	sgRNA_82035	RFXANK	114.97	1681.8/369.91/9.0004/0	114.97	189.46	0.71574	7.76E+05	1.20E+05	0.21494	0.34138	0.65862	0.68275	0.68614	True

Screen	sgRNA	gene	control_count	treatment_count	control_mean	treat_mean	LFC	control_var	adj_var	score	p.low	p.high	p.twosided	FDR	high_in_treat
	sgRNA_79422	LOC100624181	113.1	34887/95269/43469/1400.1	113.1	39178	8.4237	1.54E+09	1.16E+05	114.6	1	0	0	0	True
	sgRNA_79421	LOC100624181	140.2	32384/56805/14813/589.11	140.2	23599	7.3848	5.95E+08	1.79E+05	55.434	1	0	0	0	True
	sgRNA_79424	LOC100624181	33.649	0/0/0/0	33.649	0	-5.1147	0	10138	2.7914	0.0026237	0.99738	0.0052473	0.0068174	False
	sgRNA_79423	LOC100624181	57.951	434.37/526.17/7.7146/0	57.951	221.04	1.9133	77661	30254	0.93765	0.72369	0.27631	0.55261	0.55697	True
	sgRNA_14143	CCZ1	88.796	37010/92900/5.3645e+05/1.6185e+06	88.796	3.15E+05	11.775	6.25E+11	71407	1177.3	1	0	0	0	True
	sgRNA_14142	CCZ1	25.237	331.1/467.02/466.74/401.14	25.237	433.94	4.0511	4607.5	5686	5.42	1	4.72E-08	9.44E-08	3.28E-05	True
	sgRNA_14144	CCZ1	65.429	344.26/0/0/0	65.429	0	-6.0537	39505	38622	3.001	0.0013453	0.99865	0.0026906	0.0048392	False
	sgRNA_14141	CCZ1	73.841	417.16/1922.8/159.44/131.78	73.841	288.3	1.9507	9.10E+05	49265	0.96621	0.7351	0.2649	0.5298	0.53429	True
	sgRNA_63352	MARCO	41.127	2739.9/1339.3/393.45/16.472	41.127	866.36	4.3638	1.56E+06	15177	6.6986	1	1.67E-11	3.34E-11	1.30E-08	True
	sgRNA_63349	MARCO	127.12	2082.8/10053/1950.5/3.8757	127.12	2016.6	3.9771	2.29E+07	1.47E+05	4.9279	1	6.60E-07	1.32E-06	0.00042303	True
	sgRNA_63350	MARCO	160.77	0/0/0/0	160.77	0	-7.3378	0	2.36E+05	3.2661	0.00054521	0.99945	0.0010904	0.0048138	False
	sgRNA_63351	MARCO	40.192	0/0/0/0	40.192	0	-5.3643	0	14491	2.8487	0.0021948	0.99781	0.0043897	0.0060187	False
**Screen 1**	sgRNA_15503	LOC100736732	114.42	42960/62914/12387/1014.5	114.42	27674	7.9056	8.07E+08	2.16E+05	59.305	1	0	0	0	True
**Subset 2**	sgRNA_15502	LOC100736732	109.93	42635/94440/5884.4/393.44	109.93	24259	7.7728	2.06E+09	2.00E+05	54.052	1	0	0	0	True
	sgRNA_15501	LOC100736732	196.3	43414/1.6644e+05/21741/1049.2	196.3	32578	7.3674	6.38E+09	6.24E+05	40.987	1	0	0	0	True
	sgRNA_15504	LOC100736732	209.76	46247/1.352e+05/11800/577.79	209.76	29024	7.1055	4.23E+09	7.11E+05	34.17	1	5.72E-256	1.14E-255	2.27E-252	True
	sgRNA_35433	SLA-DMB	0	6486.8/14940/731.32/16.084	1.1217	3609.1	10.733	5.26E+07	25.339	716.74	1	0	0	0	True
	sgRNA_35432	SLA-DMB	190.69	48912/1.9643e+05/73495/14603	190.69	61203	8.3187	6.92E+09	5.90E+05	79.461	1	0	0	0	True
	sgRNA_35431	SLA-DMB	96.468	25461/62002/4333.9/258.58	96.468	14897	7.256	8.85E+08	1.54E+05	37.666	1	1.49E-306	2.98E-306	6.37E-307	True
	sgRNA_35434	SLA-DMB	235.56	5580.6/27402/8157.5/595.11	235.56	6869.1	4.86	1.55E+08	8.93E+05	7.0187	1	1.87E-12	3.74E-12	2.42E-09	True
	sgRNA_15500	LOC106509697	76.277	42968/1.665e+05/55850/8628.5	76.277	49409	9.3205	5.15E+09	97313	158.14	1	0	0	0	True
	sgRNA_15499	LOC106509697	153.68	20493/1.0634e+05/25235/1571.3	153.68	22864	7.2077	2.48E+09	3.86E+05	36.568	1	7.78E-293	1.56E-292	3.24E-289	True
	sgRNA_15498	LOC106509697	141.34	5434.1/7848.7/186.43/7.4234	141.34	2810.3	4.3038	1.57E+07	3.27E+05	4.666	1	2.57E-06	5.14E-06	0.0022198	True
	sgRNA_15497	LOC106509697	85.251	2411/11716/619.03/6.1862	85.251	1515	4.1356	3.60E+07	1.21E+05	4.1087	0.99997	3.33E-05	6.67E-05	0.002565	True
	sgRNA_82033	RFXANK	167.14	21010/65167/10936/522.11	167.14	15973	6.57	9.03E+08	4.55E+05	23.434	1	1.60E-121	3.21E-121	4.61E-118	True
	sgRNA_82036	RFXANK	59.451	8901.6/14071/605.35/18.558	59.451	4753.5	6.2974	4.79E+07	59624	19.224	1	1.97E-82	3.93E-82	5.12E-79	True
	sgRNA_82035	RFXANK	137.97	7627.6/14286/169.15/0	137.97	3898.4	4.8104	5.03E+07	3.12E+05	6.7319	1	1.40E-11	2.80E-11	1.71E-08	True
	sgRNA_82034	RFXANK	112.17	3487.7/2621.6/56.145/0	112.17	1338.9	3.5655	3.23E+06	2.08E+05	2.6917	0.99405	0.0059525	0.011905	0.013315	True
	sgRNA_35437	SLA-DMA	84.129	33160/64012/5304.2/269.72	84.129	19232	7.8197	9.18E+08	1.18E+05	55.746	1	0	0	0	True
	sgRNA_35436	SLA-DMA	42.625	7938/20953/3437.1/374.88	42.625	5687.5	7.0267	9.05E+07	31005	32.058	1	1.41E-225	2.83E-225	5.02E-222	True
	sgRNA_35438	SLA-DMA	42.625	2848.8/14199/1177.6/127.44	42.625	2013.2	5.5289	5.12E+07	31005	11.191	1	3.79E-29	7.57E-29	7.17E-26	True

Screen	sgRNA	gene	control_count	treatment_count	control_mean	treat_mean	LFC	control_var	adj_var	score	p.low	p.high	p.twosided	FDR	high_in_treat
	sgRNA_35435	SLA-DMA	10.095	0/0/0/0	10.095	0	-3.4719	0	1832.7	2.5424	0.0055041	0.9945	0.011008	0.012343	False
	sgRNA_79422	LOC100624181	135.73	35379/76727/13277/2541.3	135.73	24328	7.4752	1.15E+09	3.02E+05	44.012	1	0	0	0	True
	sgRNA_79421	LOC100624181	168.26	31877/79158/10906/948.96	168.26	21391	6.9817	1.32E+09	4.61E+05	31.26	1	1.38E-214	2.76E-214	4.80E-211	True
	sgRNA_79423	LOC100624181	69.547	1365/5122.1/601.04/30.931	69.547	983.01	3.802	6.11E+06	81154	3.2065	0.99887	0.0011262	0.0022524	0.0031108	True
	sgRNA_79424	LOC100624181	40.382	0/0/0/0	40.382	0	-5.3709	0	27880	2.9883	0.0014026	0.9986	0.0028053	0.0036283	False
	sgRNA_31072	LOC102165390	187.33	10370/15938/322.47/0	187.33	5346.3	4.8275	6.38E+07	5.69E+05	6.8375	1	6.74E-12	1.35E-11	8.32E-09	True
	sgRNA_31074	LOC102165390	44.869	1781.7/6574.2/131.72/0	44.869	956.7	4.384	1.13E+07	34294	4.9239	1	7.12E-07	1.42E-06	0.00065606	True
	sgRNA_31075	LOC102165390	130.12	3442.6/7638.8/48.947/0	130.12	1745.8	3.7358	1.45E+07	2.78E+05	3.0639	0.99817	0.0018286	0.0036572	0.0045087	True
	sgRNA_31073	LOC102165390	241.17	1058.5/2502.8/29.512/0	241.17	543.99	1.1702	1.55E+06	9.36E+05	0.31307	0.36985	0.63015	0.7397	0.74262	True
	sgRNA_14142	CCZ1	30.286	3124.6/84873/4.6896e+05/7.2322e+05	30.286	2.77E+05	13.112	1.16E+11	15841	2199.9	1	0	0	0	True
	sgRNA_14143	CCZ1	106.56	30244/51211/34137/8785.6	106.56	32190	8.2253	3.06E+08	1.88E+05	74.04	1	0	0	0	True
	sgRNA_14141	CCZ1	88.616	0/0/0/0	88.616	0	-6.4857	0	1.31E+05	3.2178	0.00064589	0.99935	0.0012918	0.002565	False
	sgRNA_14144	CCZ1	78.52	144.64/0/0/0	78.52	0	-6.3133	6973.6	1.03E+05	3.1834	0.00072777	0.99927	0.0014555	0.002581	False
	sgRNA_48312	RFXAP	207.52	86299/1.2166e+05/42502/6799.8	207.52	64400	8.2708	2.52E+09	6.96E+05	76.933	1	0	0	0	True
	sgRNA_48311	RFXAP	117.78	29456/51090/3280.9/116.3	117.78	16368	7.1066	6.04E+08	2.29E+05	33.988	1	2.85E-253	5.70E-253	1.11E-249	True
	sgRNA_48314	RFXAP	246.78	340.05/0/0/0	246.78	0	-7.9529	38545	9.79E+05	3.4972	0.00023507	0.99976	0.00047014	0.002565	False
	sgRNA_48313	RFXAP	196.3	0/0/0/0	196.3	0	-7.6243	0	6.24E+05	3.4365	0.00029463	0.99971	0.00058927	0.002565	False
	sgRNA_29333	LYPD4	47.112	1360.2/3524.7/227.46/0	47.112	793.83	4.0462	2.91E+06	37745	3.8435	0.9999	0.00010179	0.00020357	0.002565	True
	sgRNA_29335	LYPD4	142.46	4278.9/7622.6/59.024/0	142.46	2168.9	3.919	1.45E+07	3.32E+05	3.5155	0.99963	0.00036731	0.00073462	0.002565	True
	sgRNA_29334	LYPD4	79.642	0/0/0/0	79.642	0	-6.3335	0	1.06E+05	3.1875	0.00071765	0.99928	0.0014353	0.0025788	False
	sgRNA_29332	LYPD4	190.69	266.29/29.988/0/0	190.69	14.994	-3.5832	21275	5.90E+05	2.2365	0.01266	0.98734	0.02532	0.027425	False
**Screen 2**	sgRNA_01971	TMEM30A	72.115	24698/1.5051e+05/1.6502e+05/22928	72.115	87602	10.227	6.03E+09	24328	561.19	1	0	0	0	True
**Subset 1**	sgRNA_01972	TMEM30A	114.35	43593/79139/33867/3227.7	114.35	38730	8.3913	9.80E+08	55845	163.41	1	0	0	0	True
	sgRNA_01970	TMEM30A	84.478	12177/36185/25484/931.91	84.478	18831	7.7834	2.37E+08	32355	104.22	1	0	0	0	True
	sgRNA_01969	TMEM30A	61.813	7461.5/16928/5073.1/348.2	61.813	6267.3	6.6409	5.05E+07	18428	45.713	1	0	0	0	True
	sgRNA_35433	SLA-DMB	3.0907	20377/6521.1/3250.9/689.35	3.0907	4886	10.222	8.76E+07	85.589	527.8	1	0	0	0	True
	sgRNA_35432	SLA-DMB	147.32	3.6773e+05/1.5173e+05/1.2923e+05/1.1312e+05	147.32	1.40E+05	9.8874	1.75E+10	88171	472.6	1	0	0	0	True
	sgRNA_35431	SLA-DMB	76.236	35776/89192/68988/44322	76.236	56655	9.5187	6.00E+08	26890	345.03	1	0	0	0	True
	sgRNA_35434	SLA-DMB	239.01	1.0646/0/0/2.3474	239.01	0.53228	-7.2913	1.3815	2.11E+05	3.2495	0.00057814	0.99942	0.0011563	0.0038069	False
	sgRNA_15502	LOC100736732	62.843	2.0023e+05/4.6006e+05/3.2608e+05/2.5805e+05	62.843	2.92E+05	12.159	1.30E+10	18985	2119.2	1	0	0	0	True
	sgRNA_15503	LOC100736732	174.11	88784/3.0088e+05/4.2466e+05/4.2037e+05	174.11	3.61E+05	11.008	2.84E+10	1.19E+05	1044.2	1	0	0	0	True

Screen	sgRNA	gene	control_count	treatment_count	control_mean	treat_mean	LFC	control_var	adj_var	score	p.low	p.high	p.twosided	FDR	high_in_treat
	sgRNA_15504	LOC100736732	164.83	88758/27085/15781/7823.8	164.83	21433	7.014	1.59E+09	1.08E+05	64.727	1	0	0	0	True
	sgRNA_15501	LOC100736732	140.11	3650.4/142.16/0/0	140.11	71.079	-0.96916	4.28E+06	80543	1.1024	0.13515	0.86485	0.2703	0.27121	False
	sgRNA_15499	LOC106509697	55.632	21771/4149.3/1106.8/16.432	55.632	2628.1	5.5368	1.26E+08	15242	20.837	1	1.49E-96	2.99E-96	3.19E-93	True
	sgRNA_15500	LOC106509697	65.934	7690.4/2151.6/795.32/19.562	65.934	1473.5	4.4613	1.39E+07	20700	9.783	1	9.84E-23	1.97E-22	1.45E-19	True
	sgRNA_15498	LOC106509697	264.77	9755.6/3558.2/328.62/70.421	264.77	1943.4	2.8711	2.33E+07	2.54E+05	3.3323	0.99939	0.00061492	0.0012298	0.0038069	True
	sgRNA_15497	LOC106509697	92.72	0/0/0/0	92.72	0	-6.5503	0	38265	3.0769	0.0010457	0.99895	0.0020915	0.0038069	False
	sgRNA_48312	RFXAP	91.689	1.5024e+05/1.3896e+05/1.0543e+05/35571	91.689	1.22E+05	10.364	2.95E+09	37502	630.51	1	0	0	0	True
	sgRNA_48311	RFXAP	58.722	1.1462e+05/55726/36244/15697	58.722	45985	9.5887	1.94E+09	16801	354.32	1	0	0	0	True
	sgRNA_48314	RFXAP	276.1	0/0/0/0	276.1	0	-8.1143	0	2.74E+05	3.3741	0.00037025	0.99963	0.0007405	0.0038069	False
	sgRNA_48313	RFXAP	138.05	11103/1034.7/278.42/34.428	138.05	656.54	2.2415	3.66E+07	78420	1.8515	0.95349	0.046515	0.09303	0.094991	True
	sgRNA_79421	LOC100624181	97.871	1.3272e+05/2.6053e+05/1.8149e+05/1.0542e+05	97.871	1.57E+05	10.634	4.85E+09	42182	764.47	1	0	0	0	True
	sgRNA_79422	LOC100624181	205.01	98905/1.9526e+05/1.3727e+05/79457	205.01	1.18E+05	9.1629	2.73E+09	1.60E+05	294.71	1	0	0	0	True
	sgRNA_79423	LOC100624181	137.02	0/0/0/0	137.02	0	-7.1087	0	77368	3.1858	0.00072176	0.99928	0.0014435	0.0038069	False
	sgRNA_79424	LOC100624181	105.08	0/0/0/0	105.08	0	-6.729	0	47950	3.1121	0.00092865	0.99907	0.0018573	0.0038069	False
	sgRNA_35436	SLA-DMA	79.327	49367/39149/42439/34122	79.327	40794	8.9883	4.11E+07	28886	239.56	1	0	0	0	True
	sgRNA_35437	SLA-DMA	70.055	56133/16684/4678.3/446.79	70.055	10681	7.232	7.48E+08	23089	69.831	1	0	0	0	True
	sgRNA_35438	SLA-DMA	53.571	0/0/0/0	53.571	0	-5.7701	0	14240	2.9186	0.0017578	0.99824	0.0035155	0.0044886	False
	sgRNA_35435	SLA-DMA	14.423	0/0/0/0	14.423	0	-3.947	0	1343.6	2.5094	0.0060473	0.99395	0.012095	0.012746	False
	sgRNA_82036	RFXANK	48.42	22028/3195.9/1582.6/389.67	48.42	2389.3	5.5959	1.30E+08	11869	21.486	1	1.56E-102	3.11E-102	3.37E-99	True
	sgRNA_82033	RFXANK	112.29	12484/4953.1/2511.5/345.07	112.29	3732.3	5.0423	3.03E+07	54044	15.572	1	8.28E-55	1.66E-54	1.52E-51	True
	sgRNA_82034	RFXANK	145.26	0/0/0/0.78246	145.26	0	-7.1924	0.20408	85960	3.2018	0.00068277	0.99932	0.0013655	0.0038069	False
	sgRNA_82035	RFXANK	101.99	1387.1/0/0/0	101.99	0	-6.6864	6.41E+05	45437	3.1038	0.00095534	0.99904	0.0019107	0.0038069	False
	sgRNA_59197	VPS33A	76.236	11069/84121/90296/7262	76.236	47595	9.2673	2.04E+09	26890	289.78	1	0	0	0	True
	sgRNA_59198	VPS33A	161.74	3915.4/3152.1/871.77/62.597	161.74	2011.9	3.6286	3.34E+06	1.04E+05	5.7277	1	7.36E-09	1.47E-08	8.29E-06	True
	sgRNA_59196	VPS33A	41.209	0/0/0/0	41.209	0	-5.3995	0	8877.6	2.8405	0.002252	0.99775	0.0045041	0.0053133	False
	sgRNA_59195	VPS33A	94.78	2652.9/2.1377/0/0	94.78	1.0689	-5.5328	2.34E+06	39811	2.7703	0.0028003	0.9972	0.0056006	0.006328	False
	sgRNA_03161	VPS18	49.45	5357.9/6495.5/1849.7/84.506	49.45	3603.8	6.1589	8.97E+06	12328	32.012	1	5.54E-225	1.11E-224	1.40E-221	True
	sgRNA_03164	VPS18	25.755	1801.2/514.12/151.76/0	25.755	332.94	3.6417	7.77E+05	3809.8	4.9768	1	4.88E-07	9.77E-07	0.00052211	True
	sgRNA_03163	VPS18	124.66	414.11/38.479/12.552/0	124.66	25.515	-2.2446	50665	65241	1.6195	0.052665	0.94734	0.10533	0.10751	False
	sgRNA_03162	VPS18	85.508	5312.1/390.13/101.55/0	85.508	245.84	1.5127	8.59E+06	33069	0.8817	0.72247	0.27753	0.55506	0.55586	True

Screen	sgRNA	gene	control _count	treatment _count	control _mean	treat _mean	LFC	control _var	adj _var	score	p.low	p.high	p.twosided	FDR	high_in _treat
**Screen 2**	sgRNA_35433	SLA-DMB	3.9477	51183/2.0493e+05/1.3555e+05/1.4215e+05	3.9477	1.39E+05	14.776	4.02E+09	137.01	11862	1	0	0	0	True
**Subset 2**	sgRNA_35432	SLA-DMB	188.17	2.0297e+05/1.9408e+05/1.1582e+05/89069	188.17	1.55E+05	9.6779	3.24E+09	1.48E+05	402.75	1	0	0	0	True
	sgRNA_35434	SLA-DMB	305.29	73604/79767/33646/8622.7	305.29	53625	7.4519	1.17E+09	3.55E+05	89.481	1	0	0	0	True
	sgRNA_35431	SLA-DMB	97.376	78825/22549/11262/3904.7	97.376	16906	7.4251	1.36E+09	44727	79.476	1	0	0	0	True
	sgRNA_15503	LOC100736732	222.38	1.9952e+05/5.3955e+05/4.3239e+05/3.8788e+05	222.38	4.10E+05	10.842	2.07E+10	2.00E+05	916.85	1	0	0	0	True
	sgRNA_15502	LOC100736732	80.269	55378/66847/28577/9694.2	80.269	41978	9.0127	6.73E+08	31515	236.01	1	0	0	0	True
	sgRNA_15504	LOC100736732	210.54	58610/41836/14457/12780	210.54	28147	7.0559	5.13E+08	1.81E+05	65.664	1	0	0	0	True
	sgRNA_15501	LOC100736732	178.96	68696/9859/3786.6/1814	178.96	6822.8	5.2448	1.29E+09	1.35E+05	18.095	1	2.53E-73	5.06E-73	3.67E-70	True
	sgRNA_14143	CCZ1	135.54	1.6738e+05/2.3364e+06/4.3581e+06/5.9859e+06	135.54	3.35E+06	14.581	6.37E+12	81447	11728	1	0	0	0	True
	sgRNA_14141	CCZ1	90.796	6032.5/79054/77590/44663	90.796	61126	9.3792	1.30E+09	39401	307.49	1	0	0	0	True
	sgRNA_14142	CCZ1	31.581	8290.7/24920/9467.3/1402	31.581	8879	8.0904	1.05E+08	5818.4	115.99	1	0	0	0	True
	sgRNA_14144	CCZ1	203.96	0/0/0/0	203.96	0	-7.6792	0	1.71E+05	3.3765	0.00036704	0.99963	0.00073408	0.0022443	False
	sgRNA_48311	RFXAP	75.006	35558/46917/30299/13461	75.006	32928	8.7591	1.96E+08	27870	196.79	1	0	0	0	True
	sgRNA_48312	RFXAP	117.11	65533/16780/4698.7/914.69	117.11	10739	6.5067	1.06E+09	62497	42.49	1	0	0	0	True
	sgRNA_48313	RFXAP	176.33	17220/7627.7/2500.9/285.94	176.33	5064.3	4.8361	6.12E+07	1.31E+05	13.493	1	1.25E-41	2.51E-41	1.61E-38	True
	sgRNA_48314	RFXAP	352.66	0/0/0/0	352.66	0	-8.4662	0	4.61E+05	3.5177	0.00021762	0.99978	0.00043524	0.0022443	False
	sgRNA_82034	RFXANK	185.54	5412.9/59352/38531/5259.1	185.54	21972	6.8801	7.42E+08	1.44E+05	57.428	1	0	0	0	True
	sgRNA_82033	RFXANK	143.43	47914/12504/5460.5/1480.4	143.43	8982.2	5.9588	5.32E+08	90252	29.421	1	2.14E-190	4.28E-190	3.92E-187	True
	sgRNA_82036	RFXANK	61.847	2890.2/2138.7/1568.6/298.24	61.847	1853.7	4.8832	1.22E+06	19649	12.783	1	1.53E-37	3.06E-37	1.90E-34	True
	sgRNA_82035	RFXANK	130.27	0/0/0/0	130.27	0	-7.0364	0	75804	3.2573	0.0005624	0.99944	0.0011248	0.0022443	False
	sgRNA_01972	TMEM30A	146.06	26791/51585/35665/4545.8	146.06	31228	7.7303	3.89E+08	93276	101.77	1	0	0	0	True
	sgRNA_01971	TMEM30A	92.112	8430.7/3628.1/1156.6/3.0746	92.112	2392.3	4.6839	1.51E+07	40442	11.438	1	1.99E-30	3.98E-30	2.42E-27	True
	sgRNA_01970	TMEM30A	107.9	1878.4/3166.1/1404.6/0	107.9	1641.5	3.9148	1.71E+06	53874	6.6074	1	2.88E-11	5.76E-11	2.91E-08	True
	sgRNA_01969	TMEM30A	78.953	0/0/0/0	78.953	0	-6.3211	0	30585	3.1198	0.00090476	0.9991	0.0018095	0.0024587	False
	sgRNA_59197	VPS33A	97.376	3682.7/3888.7/2189.5/24.597	97.376	2936.1	4.9	3.50E+06	44727	13.423	1	3.28E-41	6.57E-41	4.18E-38	True
	sgRNA_59196	VPS33A	52.635	4757.2/2085.9/1259.6/222.91	52.635	1672.8	4.9638	3.99E+06	14672	13.376	1	6.28E-41	1.26E-40	7.93E-38	True
	sgRNA_59195	VPS33A	121.06	5023.8/2563/293.6/0	121.06	1428.3	3.5496	5.85E+06	66368	5.0743	1	2.86E-07	5.71E-07	0.00026027	True
	sgRNA_59198	VPS33A	206.59	2136.7/244.46/0/0	206.59	122.23	-0.75244	1.37E+06	1.75E+05	0.99975	0.15871	0.84129	0.31743	0.31854	False
	sgRNA_35435	SLA-DMA	18.422	24679/10911/4928.4/1299	18.422	7919.7	8.6718	1.14E+08	2194.9	168.65	1	0	0	0	True
	sgRNA_35438	SLA-DMA	68.426	0/0/0/0	68.426	0	-6.1174	0	23599	3.0797	0.0010362	0.99896	0.0020724	0.0026492	False
	sgRNA_35437	SLA-DMA	89.48	25553/1174.9/155.08/35.358	89.48	664.99	2.8798	2.07E+08	38372	2.938	0.99756	0.0024434	0.0048867	0.0052786	True

Screen	sgRNA	gene	control _count	treatment _count	control _mean	treat _mean	LFC	control _var	adj _var	score	p.low	p.high	p.twosided	FDR	high_in _treat
	sgRNA_35436	SLA-DMA	101.32	11884/481.37/248.61/96.85	101.32	364.99	1.8387	4.43E+07	48068	1.2026	0.83104	0.16896	0.33793	0.33906	True
	sgRNA_15500	LOC106509697	84.217	1.1659e+05/6.8483e+05/7.6903e+05/3.3031e+05	84.217	5.08E+05	12.54	9.47E+10	34380	2737	1	0	0	0	True
	sgRNA_15499	LOC106509697	71.058	40318/17287/5771.3/3214.5	71.058	11529	7.3221	3.21E+08	25269	72.081	1	0	0	0	True
	sgRNA_15497	LOC106509697	118.43	0/0/0.59193/0	118.43	0	-6.9	0.11679	63776	3.2315	0.00061573	0.99938	0.0012315	0.0022443	False
	sgRNA_15498	LOC106509697	338.18	508.46/199.23/221.38/84.551	338.18	210.3	-0.68275	34985	4.27E+05	0.94628	0.172	0.828	0.344	0.34514	False
	sgRNA_79422	LOC100624181	261.86	63811/38157/28673/7821.8	261.86	33415	6.9901	5.41E+08	2.69E+05	63.942	1	0	0	0	True
	sgRNA_79421	LOC100624181	125.01	52435/12654/3503.6/1109.9	125.01	8078.6	6.0027	6.86E+08	70343	29.988	1	1.03E-197	2.05E-197	1.90E-194	True
	sgRNA_79423	LOC100624181	175.01	0/0/0/0	175.01	0	-7.4595	0	1.29E+05	3.3362	0.00042466	0.99958	0.00084933	0.0022443	False
	sgRNA_79424	LOC100624181	134.22	0/0/0/0	134.22	0	-7.0792	0	80020	3.2653	0.00054666	0.99945	0.0010933	0.0022443	False

sgRNA: sgRNA ID; gene: targeting gene; control_count: normalized read counts in control samples; treatment_count: normalized read counts in treatment samples; control_mean: mean read counts in control samples; treat_mean: mean read counts in treatment samples; LFC: log fold change of sgRNA; control_var: raw variance in control samples; adj_var: adjusted variance in control samples; score: score of this sgRNA; p.low: p-value (lower tail); p.high: p-value (higher tail); p.twosided: p-value (two sided); FDR: false discovery rate; high_in_treatment: whether the abundance is higher in treatment samples [1].

Table S4. Selected sgRNA sequences for the generation of WSL knockout cells.

sgRNA_ID	Target sequence	Target gene
sgRNA_35436	GTGGGCCCGATTCCTTCCAC	SLA-DMA
sgRNA_35433	TCAGCGCTCCAAATTCACAA	SLA-DMB
sgRNA_48312	CAGTGAGACCACAAGCCAGG	RFXAP
sgRNA_15502	CAGAAGACAAAGTCGTACTG	LOC100736732 (CIITA)

Table S5. Primers used for PCR, sequencing and qPCR.

Primer	Sequence 5' to 3'	Method	Aim
HU6-SF	ATAATTTCTTGGGTAGTTTGCAG	Sequencing	Analysis of plasmid clones
M13 Rev (-24)	AACAGCTATGACCATG	Sequencing	Analysis of plasmid clones
X330GRR-F2	ATGCTTACCGTAACTTGAAAG	PCR	Confirmation of sgRNA gene integration into WSL cells
X330GRR-R2	ATTTGTCTGCAGAATTGGCG	PCR and sequencing	
SLADMA-552F	ATGCGCCTTTGTCTCCAC	PCR and sequencing	Confirmation of SLA-DMA knockout
SLADMA-1278R	CATCGCTGAGGTGTTCAC	PCR and sequencing	
SLADMB-4475F	ATCTCAAACCCTCTGCCAG	PCR	Confirmation of SLA-DMB knockout
SLADMB- 4991R	CTCAGAGACTCCAGTACCC	PCR and sequencing	
CIITA-19351F	CTCTGCCGATGGGAACTG	PCR	Confirmation of CIITA knockout
CIITA-20278R	CAAGAGTCAGGAGAGAGAGC	PCR and sequencing	
RFXAP-29005F	GAGCTCAGATGCACTCAG	PCR	Confirmation of RFXAP knockout
RFXAP-29952R	AGATCCCAACTGATACCTCC	PCR and sequencing	
CMV promotor-F	AATGGGAGTTTGTTTTGGCACC	PCR and sequencing	Confirmation of transgene insertion
PLVX-IRES-R	AAAGACGGCAATATGGTGG	PCR and sequencing	
AKB646L-408F	GCTGTATCTCTATCCTGAAAGC	qPCR	Quantification of ASFV genome copy numbers
AKB646L-507R	CTCATCAACACCGAGATTGG	qPCR	
AKB646L-460P	FAM-TGGCGTTAACAACATGTCCGAAC-TAMRA	qPCR	
ACT-CP-F	CCTGACCGACTACCTCATGAAG	qPCR	Standardization of ASFV genome copy numbers
ACT-CP-R	CATCTCCTGCTCGAAGTCCAG	qPCR	
ACT-CP-P	Hex-CGTGGTGGTGAAGCTGTAGCCC-BHQ1	qPCR	

Table S6. List of identified proteins in WSL and WSL knockout cells. MS-analysis of whole cell lysates of WSL, WSL SLA-DMA_{KO} (11), WSL SLA-DMB_{KO} (9), WSL $RFXAP_{KO}$ (6), and WSL $CIITA_{KO}$ cells (1) was performed on three independently treated samples on a proteome wide level. ***The data can be found in a separate file (Table S6.xlsx).***

Table S7. Oligonucleotides used for cloning of selected sgRNA sequences into vector pX330A-1x4neoRA as well as for cloning of StrepII- and Myc-tag.

Oligonucleotide	Sequence 5' to 3'
SLA-DMAgR2-F	CACCGTGGGCCCGATTCCTTCCAC
SLA-DMAgR2-R	AAACGTGGAAGGAATCGGGCCCAC
SLA-DMBgR3-F	CACCGTCAGCGCTCCAAATTCACAA
SLA-DMBgR3-R	AAACTTGTGAATTTGGAGCGCTGAC
MHCIITAgR2-F	CACCGCAGAAGACAAAGTCGTACTG
MHCIITAgR2-R	AAACCAGTACGACTTTGTCTTCTGC
RFXAPgR2-F	CACCGCAGTGAGACCACAAGCCAGG
RFXAPgR2-R	AAACCCTGGCTTGTGGTCTCACTGC
StrepII-tag-SLA-DMA-F	GCCTGGTCACATCCTCAGTTCGAGAAGTAGC
StrepII-tag-SLA-DMB-F	TCCTGGTCACATCCTCAGTTCGAGAAGTAGC
StrepII-tag-AR	GGCCGCTACTTCTCGAACTGAGGATGTGACC
Lmyc-tag-SLA-DMA-F	GCCTTGGAGCAGAAGCTCATCTCTGAGGAAGATCTCTAGC
Lmyc-tag-SLA-DMB-F	TCCTTGGAGCAGAAGCTCATCTCTGAGGAAGATCTCTAGC
Lmyc-tag-R	GGCCGCTAGAGATCTTCCTCAGAGATGAGCTTCTGCTCCA

Table S8. Statistical analysis of plating efficiency of ASFV Armenia grown on WSL, WSL knockout and WSL knockout / knockin cells. Plating efficiency was calculated by counting ASFV-infected cells or plaques in three independent experiments (n = 6). Relative (%) titers were determined by comparison to the mean titer on WSL cells in the same experiment (100%). Significant differences between indicated cell lines was calculated by ordinary one-way ANOVA followed by Tukey's multiple comparison test (GraphPad Prism; Version 9).

Number of families			1				
Number of comparisons per family			120				
Alpha			0,05				
Tukey's multiple comparisons test			Mean Diff,	95,00% CI of diff,	Below threshold?	Summary	Adjusted p value
WSL	vs	WSL-GFP$_{KI}$	-33	-90,74 to 24,75	No	ns	0,7917
WSL	vs	WSL-DMA$_{KI}$	-1,176	-55,35 to 53,00	No	ns	>0,9999
WSL	vs	WSL-DMA-Myc$_{KI}$	34,78	-28,48 to 98,04	No	ns	0,8337
WSL	vs	WSL-DMA-Strep$_{KI}$	18,7	-35,47 to 72,87	No	ns	0,9968
WSL	vs	WSL-DMB$_{KI}$	-56,77	-120,0 to 6,492	No	ns	0,1262
WSL	vs	WSL-DMB-Myc$_{KI}$	33,27	-20,91 to 87,44	No	ns	0,6966
WSL	vs	SLA-DMA$_{KO}$	99,49	45,32 to 153,7	Yes	****	<0,0001
WSL	vs	SLA-DMA$_{KO}$-GFP$_{KI}$	99,06	44,88 to 153,2	Yes	****	<0,0001
WSL	vs	SLA-DMA$_{KO}$-DMA$_{KI}$	23,4	-30,77 to 77,58	No	ns	0,9719
WSL	vs	SLA-DMA$_{KO}$-DMA-Myc$_{KI}$	57,65	3,477 to 111,8	Yes	*	0,0263
WSL	vs	SLA-DMA$_{KO}$-DMA-Strep$_{KI}$	21,35	-32,82 to 75,52	No	ns	0,9878
WSL	vs	SLA-DMB$_{KO}$	98,59	44,41 to 152,8	Yes	****	<0,0001
WSL	vs	SLA-DMB$_{KO}$-GFP$_{KI}$	98,5	44,33 to 152,7	Yes	****	<0,0001
WSL	vs	SLA-DMB$_{KO}$-DMB$_{KI}$	22,54	-31,63 to 76,71	No	ns	0,9799
WSL	vs	SLA-DMB$_{KO}$-DMB-Myc$_{KI}$	37,12	-17,06 to 91,29	No	ns	0,5203
WSL-GFP$_{KI}$	vs	WSL-DMA$_{KI}$	31,82	-28,19 to 91,83	No	ns	0,8676
WSL-GFP$_{KI}$	vs	WSL-DMA-Myc$_{KI}$	67,78	-0,5462 to 136,1	No	ns	0,054
WSL-GFP$_{KI}$	vs	WSL-DMA-Strep$_{KI}$	51,7	-8,313 to 111,7	No	ns	0,1688
WSL-GFP$_{KI}$	vs	WSL-DMB$_{KI}$	-23,77	-92,10 to 44,56	No	ns	0,9965
WSL-GFP$_{KI}$	vs	WSL-DMB-Myc$_{KI}$	66,26	6,251 to 126,3	Yes	*	0,0173
WSL-GFP$_{KI}$	vs	SLA-DMA$_{KO}$	132,5	72,47 to 192,5	Yes	****	<0,0001
WSL-GFP$_{KI}$	vs	SLA-DMA$_{KO}$-GFP$_{KI}$	132,1	72,04 to 192,1	Yes	****	<0,0001
WSL-GFP$_{KI}$	vs	SLA-DMA$_{KO}$-DMA$_{KI}$	56,4	-3,611 to 116,4	No	ns	0,0874
WSL-GFP$_{KI}$	vs	SLA-DMA$_{KO}$-DMA-Myc$_{KI}$	90,65	30,63 to 150,7	Yes	***	0,0001
WSL-GFP$_{KI}$	vs	SLA-DMA$_{KO}$-DMA-Strep$_{KI}$	54,35	-5,665 to 114,4	No	ns	0,1177
WSL-GFP$_{KI}$	vs	SLA-DMB$_{KO}$	131,6	71,57 to 191,6	Yes	****	<0,0001
WSL-GFP$_{KI}$	vs	SLA-DMB$_{KO}$-GFP$_{KI}$	131,5	71,49 to 191,5	Yes	****	<0,0001
WSL-GFP$_{KI}$	vs	SLA-DMB$_{KO}$-DMB$_{KI}$	55,54	-4,477 to 115,5	No	ns	0,0993
WSL-GFP$_{KI}$	vs	SLA-DMB$_{KO}$-DMB-Myc$_{KI}$	70,11	10,10 to 130,1	Yes	**	0,0086
WSL-DMA$_{KI}$	vs	SLA-DMA$_{KO}$-DMA$_{KI}$	24,58	-32,00 to 81,16	No	ns	0,9706
WSL-DMA-Myc$_{KI}$	vs	SLA-DMA$_{KO}$-DMA-Myc$_{KI}$	22,86	-42,47 to 88,20	No	ns	0,9963
WSL-DMA-Strep$_{KI}$	vs	SLA-DMA$_{KO}$-DMA-Strep$_{KI}$	2,648	-53,93 to 59,23	No	ns	>0,9999
WSL-DMB$_{KI}$	vs	SLA-DMB$_{KO}$-DMB$_{KI}$	79,3	13,97 to 144,6	Yes	**	0,0051
WSL-DMB-Myc$_{KI}$	vs	SLA-DMB$_{KO}$-DMB-Myc$_{KI}$	3,85	-52,73 to 60,43	No	ns	>0,9999
SLA-DMA$_{KO}$	vs	SLA-DMB$_{KO}$-GFP	-0,432	-57,01 to 56,15	No	ns	>0,9999
SLA-DMA$_{KO}$	vs	SLA-DMA$_{KO}$-DMA	-76,08	-132,7 to -19,50	Yes	**	0,0011
SLA-DMA$_{KO}$	vs	SLA-DMA$_{KO}$-DMA-Myc$_{KI}$	-41,84	-98,42 to 14,74	No	ns	0,3899
SLA-DMA$_{KO}$	vs	SLA-DMA$_{KO}$-DMA-Strep$_{KI}$	-78,14	-134,7 to -21,56	Yes	***	0,0007
SLA-DMB$_{KO}$	vs	SLA-DMB$_{KO}$-GFP$_{KI}$	-0,084	-56,66 to 56,50	No	ns	>0,9999
SLA-DMB$_{KO}$	vs	SLA-DMB$_{KO}$-DMB$_{KI}$	-76,05	-132,6 to -19,47	Yes	**	0,0011
SLA-DMB$_{KO}$	vs	SLA-DMB$_{KO}$-DMB-Myc$_{KI}$	-61,47	-118,1 to -4,890	Yes	*	0,0209

KI: knockin; KO: knockout; vs: versus

Table S9. Statistical analysis of plating efficiency of ASFV Kenya grown on WSL, WSL knockout and WSL knockout / knockin cells. Plating efficiency was calculated by counting ASFV-infected cells or plaques in three independent experiments (n = 6). Relative (%) titers were determined by comparison to the mean titer on WSL cells in the same experiment (100 %). Significant differences between indicated cell lines was calculated by ordinary one-way ANOVA followed by Tukey's multiple comparison test (GraphPad Prism; Version 9).

Number of families			1				
Number of comparisons per family			120				
Alpha			0,05				
Tukey's multiple comparisons test			Mean Diff,	95,00% CI of diff,	Below threshold?	Summary	Adjusted p value
WSL	vs	WSL-GFP$_{KI}$	-5,322	-58,91 to 48,27	No	ns	>0,9999
WSL	vs	WSL-DMA$_{KI}$	8,446	-41,82 to 58,72	No	ns	>0,9999
WSL	vs	WSL-DMA-Myc$_{KI}$	29,87	-28,84 to 88,57	No	ns	0,8996
WSL	vs	WSL-DMA-Strep$_{KI}$	13,95	-36,32 to 64,22	No	ns	0,9997
WSL	vs	WSL-DMB$_{KI}$	-24,08	-82,79 to 34,62	No	ns	0,9823
WSL	vs	WSL-DMB-Myc$_{KI}$	36,55	-13,72 to 86,82	No	ns	0,4183
WSL	vs	SLA-DMA$_{KO}$	98,94	48,67 to 149,2	Yes	****	<0,0001
WSL	vs	SLA-DMA$_{KO}$-GFP$_{KI}$	98,41	48,14 to 148,7	Yes	****	<0,0001
WSL	vs	SLA-DMA$_{KO}$-DMA$_{KI}$	13,72	-36,55 to 63,99	No	ns	0,9998
WSL	vs	SLA-DMA$_{KO}$-DMA-Myc$_{KI}$	49,62	-0,6523 to 99,89	No	ns	0,0566
WSL	vs	SLA-DMA$_{KO}$-DMA-Strep$_{KI}$	9,832	-40,44 to 60,10	No	ns	>0,9999
WSL	vs	SLA-DMB$_{KO}$	98,92	48,65 to 149,2	Yes	****	<0,0001
WSL	vs	SLA-DMB$_{KO}$-GFP$_{KI}$	98,97	48,70 to 149,2	Yes	****	<0,0001
WSL	vs	SLA-DMB$_{KO}$-DMB$_{KI}$	6,436	-43,83 to 56,71	No	ns	>0,9999
WSL	vs	SLA-DMB$_{KO}$-DMB-Myc$_{KI}$	43,79	-6,476 to 94,06	No	ns	0,1563
WSL-GFP$_{KI}$	vs	WSL-DMA$_{KI}$	13,77	-41,92 to 69,46	No	ns	>0,9999
WSL-GFP$_{KI}$	vs	WSL-DMA-Myc$_{KI}$	35,19	-28,22 to 98,60	No	ns	0,8241
WSL-GFP$_{KI}$	vs	WSL-DMA-Strep$_{KI}$	19,27	-36,42 to 74,96	No	ns	0,9967
WSL-GFP$_{KI}$	vs	WSL-DMB$_{KI}$	-18,76	-82,17 to 44,65	No	ns	0,9994
WSL-GFP$_{KI}$	vs	WSL-DMB-Myc$_{KI}$	41,88	-13,81 to 97,57	No	ns	0,3621
WSL-GFP$_{KI}$	vs	SLA-DMA$_{KO}$	104,3	48,57 to 159,9	Yes	****	<0,0001
WSL-GFP$_{KI}$	vs	SLA-DMA$_{KO}$-GFP$_{KI}$	103,7	48,04 to 159,4	Yes	****	<0,0001
WSL-GFP$_{KI}$	vs	SLA-DMA$_{KO}$-DMA$_{KI}$	19,04	-36,65 to 74,73	No	ns	0,9971
WSL-GFP$_{KI}$	vs	SLA-DMA$_{KO}$-DMA-Myc$_{KI}$	54,94	-0,7501 to 110,6	No	ns	0,0569
WSL-GFP$_{KI}$	vs	SLA-DMA$_{KO}$-DMA-Strep$_{KI}$	15,15	-40,54 to 70,85	No	ns	0,9998
WSL-GFP$_{KI}$	vs	SLA-DMB$_{KO}$	104,2	48,55 to 159,9	Yes	****	<0,0001
WSL-GFP$_{KI}$	vs	SLA-DMB$_{KO}$-GFP$_{KI}$	104,3	48,60 to 160,0	Yes	****	<0,0001
WSL-GFP$_{KI}$	vs	SLA-DMB$_{KO}$-DMB$_{KI}$	11,76	-43,93 to 67,45	No	ns	>0,9999
WSL-GFP$_{KI}$	vs	SLA-DMB$_{KO}$-DMB-Myc$_{KI}$	49,12	-6,574 to 104,8	No	ns	0,1433
WSL-DMA$_{KI}$	vs	SLA-DMA$_{KO}$-DMA$_{KI}$	5,272	-47,23 to 57,78	No	ns	>0,9999
WSL-DMA-Myc$_{KI}$	vs	SLA-DMA$_{KO}$-DMA-Myc$_{KI}$	19,75	-40,88 to 80,38	No	ns	0,9983
WSL-DMA-Strep$_{KI}$	vs	SLA-DMA$_{KO}$-DMA-Strep$_{KI}$	-4,118	-56,62 to 48,39	No	ns	>0,9999
WSL-DMB$_{KI}$	vs	SLA-DMB$_{KO}$-DMB$_{KI}$	30,52	-30,11 to 91,15	No	ns	0,9068
WSL-DMB-Myc$_{KI}$	vs	SLA-DMB$_{KO}$-DMB-Myc$_{KI}$	7,24	-45,27 to 59,75	No	ns	>0,9999
SLA-DMA$_{KO}$	vs	SLA-DMB$_{KO}$-GFP	-0,53	-53,04 to 51,98	No	ns	>0,9999
SLA-DMA$_{KO}$	vs	SLA-DMA$_{KO}$-DMA	-85,22	-137,7 to -32,71	Yes	****	<0,0001
SLA-DMA$_{KO}$	vs	SLA-DMA$_{KO}$-DMA-Myc$_{KI}$	-49,32	-101,8 to 3,188	No	ns	0,0879
SLA-DMA$_{KO}$	vs	SLA-DMA$_{KO}$-DMA-Strep$_{KI}$	-89,1	-141,6 to -36,60	Yes	****	<0,0001
SLA-DMB$_{KO}$	vs	SLA-DMB$_{KO}$-GFP$_{KI}$	0,046	-52,46 to 52,55	No	ns	>0,9999
SLA-DMB$_{KO}$	vs	SLA-DMB$_{KO}$-DMB$_{KI}$	-92,48	-145,0 to -39,98	Yes	****	<0,0001
SLA-DMB$_{KO}$	vs	SLA-DMB$_{KO}$-DMB-Myc$_{KI}$	-55,13	-107,6 to -2,620	Yes	*	0,0305

KI: knockin; KO: knockout; vs: versus

Table S10. Statistical analysis of plaque sizes of ASFV Armenia grown on WSL, WSL knockout and WSL knockout / knockin cells. For the determination of plaque sizes, areas of fifty plaques per cell line from three independent experiments (n = 150) were measured. Significant differences were calculated by Kruskal-Wallis test followed by Dunn's multiple comparison test (GraphPad Prism; Version 9).

Number of families			1			
Number of comparisons per family			120			
Alpha			0,05			
Dunn's multiple comparisons test			Mean rank diff,	Significant?	Summary	Adjusted p value
WSL	vs	WSL-GFP$_{KI}$	-47,02	No	ns	>0,9999
WSL	vs	WSL-DMA$_{KI}$	-113,1	No	ns	>0,9999
WSL	vs	WSL-DMA-Myc$_{KI}$	87,33	No	ns	>0,9999
WSL	vs	WSL-DMA-Strep$_{KI}$	-226,4	No	ns	0,2977
WSL	vs	WSL-DMB$_{KI}$	-249,5	No	ns	0,1024
WSL	vs	WSL-DMB-Myc$_{KI}$	-111,2	No	ns	>0,9999
WSL	vs	SLA-DMA$_{KO}$	1139	Yes	****	<0,0001
WSL	vs	SLA-DMA$_{KO}$-GFP$_{KI}$	1111	Yes	****	<0,0001
WSL	vs	SLA-DMA$_{KO}$-DMA$_{KI}$	-186,7	No	ns	>0,9999
WSL	vs	SLA-DMA$_{KO}$-DMA-Myc$_{KI}$	203	No	ns	0,7985
WSL	vs	SLA-DMA$_{KO}$-DMA-Strep$_{KI}$	31,03	No	ns	>0,9999
WSL	vs	SLA-DMB$_{KO}$	1114	Yes	****	<0,0001
WSL	vs	SLA-DMB$_{KO}$-GFP$_{KI}$	1062	Yes	****	<0,0001
WSL	vs	SLA-DMB$_{KO}$-DMB$_{KI}$	-365,7	Yes	***	0,0001
WSL	vs	SLA-DMB$_{KO}$-DMB-Myc$_{KI}$	-185,7	No	ns	>0,9999
WSL-GFP$_{KI}$	vs	WSL-DMA$_{KI}$	66,08	No	ns	>0,9999
WSL-GFP$_{KI}$	vs	WSL-DMA-Myc$_{KI}$	-134,3	No	ns	>0,9999
WSL-GFP$_{KI}$	vs	WSL-DMA-Strep$_{KI}$	179,4	No	ns	>0,9999
WSL-GFP$_{KI}$	vs	WSL-DMB$_{KI}$	202,5	No	ns	0,8161
WSL-GFP$_{KI}$	vs	WSL-DMB-Myc$_{KI}$	64,18	No	ns	>0,9999
WSL-GFP$_{KI}$	vs	SLA-DMA$_{KO}$	1186	Yes	****	<0,0001
WSL-GFP$_{KI}$	vs	SLA-DMA$_{KO}$-GFP$_{KI}$	1158	Yes	****	<0,0001
WSL-GFP$_{KI}$	vs	SLA-DMA$_{KO}$-DMA$_{KI}$	-139,7	No	ns	>0,9999
WSL-GFP$_{KI}$	vs	SLA-DMA$_{KO}$-DMA-Myc$_{KI}$	250	No	ns	0,0998
WSL-GFP$_{KI}$	vs	SLA-DMA$_{KO}$-DMA-Strep$_{KI}$	78,05	No	ns	>0,9999
WSL-GFP$_{KI}$	vs	SLA-DMB$_{KO}$	1161	Yes	****	<0,0001
WSL-GFP$_{KI}$	vs	SLA-DMB$_{KO}$-GFP$_{KI}$	1109	Yes	****	<0,0001
WSL-GFP$_{KI}$	vs	SLA-DMB$_{KO}$-DMB$_{KI}$	-318,6	Yes	**	0,0025
WSL-GFP$_{KI}$	vs	SLA-DMB$_{KO}$-DMB-Myc$_{KI}$	-138,6	No	ns	>0,9999
WSL-DMA$_{KI}$	vs	SLA-DMA$_{KO}$-DMA$_{KI}$	-73,59	No	ns	>0,9999
WSL-DMA-Myc$_{KI}$	vs	SLA-DMA$_{KO}$-DMA-Myc$_{KI}$	115,7	No	ns	>0,9999
WSL-DMA-Strep$_{KI}$	vs	SLA-DMA$_{KO}$-DMA-Strep$_{KI}$	257,4	No	ns	0,1547
WSL-DMB$_{KI}$	vs	SLA-DMB$_{KO}$-DMB$_{KI}$	-116,2	No	ns	>0,9999
WSL-DMB-Myc$_{KI}$	vs	SLA-DMB$_{KO}$-DMB-Myc$_{KI}$	-74,46	No	ns	>0,9999
SLA-DMA$_{KO}$	vs	SLA-DMB$_{KO}$-GFP	-76,94	No	ns	>0,9999
SLA-DMA$_{KO}$	vs	SLA-DMA$_{KO}$-DMA	-1326	Yes	****	<0,0001
SLA-DMA$_{KO}$	vs	SLA-DMA$_{KO}$-DMA-Myc$_{KI}$	-935,8	Yes	****	<0,0001
SLA-DMA$_{KO}$	vs	SLA-DMA$_{KO}$-DMA-Strep$_{KI}$	-1108	Yes	****	<0,0001
SLA-DMB$_{KO}$	vs	SLA-DMB$_{KO}$-GFP$_{KI}$	-52	No	ns	>0,9999
SLA-DMB$_{KO}$	vs	SLA-DMB$_{KO}$-DMB$_{KI}$	-1480	Yes	****	<0,0001
SLA-DMB$_{KO}$	vs	SLA-DMB$_{KO}$-DMB-Myc$_{KI}$	-1300	Yes	****	<0,0001

KI: knockin; KO: knockout; vs: versus

Table S11. Statistical analysis of plaque sizes of ASFV Kenya grown on WSL, WSL knockout and WSL knockout / knockin cells. For the determination of plaque sizes, areas of fifty plaques per cell line from three independent experiments (n = 150) were measured. Significant differences were calculated by Kruskal-Wallis test followed by Dunn's multiple comparison test (GraphPad Prism; Version 9).

Number of families			1			
Number of comparisons per family			120			
Alpha			0,05			
Dunn's multiple comparisons test			Mean rank diff,	Significant?	Summary	Adjusted p value
WSL	vs	WSL-GFP$_{KI}$	-6,47	No	ns	>0,9999
WSL	vs	WSL-DMA$_{KI}$	-161,9	No	ns	>0,9999
WSL	vs	WSL-DMA-Myc$_{KI}$	239	No	ns	0,2611
WSL	vs	WSL-DMA-Strep$_{KI}$	-0,6167	No	ns	>0,9999
WSL	vs	WSL-DMB$_{KI}$	158,7	No	ns	>0,9999
WSL	vs	WSL-DMB-Myc$_{KI}$	208,6	No	ns	0,8954
WSL	vs	SLA-DMA$_{KO}$	1208	Yes	****	<0,0001
WSL	vs	SLA-DMA$_{KO}$-GFP$_{KI}$	1094	Yes	****	<0,0001
WSL	vs	SLA-DMA$_{KO}$-DMA$_{KI}$	-421,5	Yes	****	<0,0001
WSL	vs	SLA-DMA$_{KO}$-DMA-Myc$_{KI}$	-228,5	No	ns	0,4056
WSL	vs	SLA-DMA$_{KO}$-DMA-Strep$_{KI}$	-388,6	Yes	****	<0,0001
WSL	vs	SLA-DMB$_{KO}$	1223	Yes	****	<0,0001
WSL	vs	SLA-DMB$_{KO}$-GFP$_{KI}$	1204	Yes	****	<0,0001
WSL	vs	SLA-DMB$_{KO}$-DMB$_{KI}$	-382,1	Yes	***	0,0001
WSL	vs	SLA-DMB$_{KO}$-DMB-Myc$_{KI}$	137,3	No	ns	>0,9999
WSL-GFP$_{KI}$	vs	WSL-DMA$_{KI}$	-155,4	No	ns	>0,9999
WSL-GFP$_{KI}$	vs	WSL-DMA-Myc$_{KI}$	245,5	No	ns	0,1972
WSL-GFP$_{KI}$	vs	WSL-DMA-Strep$_{KI}$	5,853	No	ns	>0,9999
WSL-GFP$_{KI}$	vs	WSL-DMB$_{KI}$	165,1	No	ns	>0,9999
WSL-GFP$_{KI}$	vs	WSL-DMB-Myc$_{KI}$	215,1	No	ns	0,6968
WSL-GFP$_{KI}$	vs	SLA-DMA$_{KO}$	1215	Yes	****	<0,0001
WSL-GFP$_{KI}$	vs	SLA-DMA$_{KO}$-GFP$_{KI}$	1101	Yes	****	<0,0001
WSL-GFP$_{KI}$	vs	SLA-DMA$_{KO}$-DMA$_{KI}$	-415	Yes	****	<0,0001
WSL-GFP$_{KI}$	vs	SLA-DMA$_{KO}$-DMA-Myc$_{KI}$	-222	No	ns	0,5281
WSL-GFP$_{KI}$	vs	SLA-DMA$_{KO}$-DMA-Strep$_{KI}$	-382,1	Yes	***	0,0001
WSL-GFP$_{KI}$	vs	SLA-DMB$_{KO}$	1229	Yes	****	<0,0001
WSL-GFP$_{KI}$	vs	SLA-DMB$_{KO}$-GFP$_{KI}$	1211	Yes	****	<0,0001
WSL-GFP$_{KI}$	vs	SLA-DMB$_{KO}$-DMB$_{KI}$	-375,6	Yes	***	0,0002
WSL-GFP$_{KI}$	vs	SLA-DMB$_{KO}$-DMB-Myc$_{KI}$	143,8	No	ns	>0,9999
WSL-DMA$_{KI}$	vs	SLA-DMA$_{KO}$-DMA$_{KI}$	-259,6	No	ns	0,2212
WSL-DMA-Myc$_{KI}$	vs	SLA-DMA$_{KO}$-DMA-Myc$_{KI}$	-467,5	Yes	****	<0,0001
WSL-DMA-Strep$_{KI}$	vs	SLA-DMA$_{KO}$-DMA-Strep$_{KI}$	-388	Yes	***	0,0004
WSL-DMB$_{KI}$	vs	SLA-DMB$_{KO}$-DMB$_{KI}$	-540,7	Yes	****	<0,0001
WSL-DMB-Myc$_{KI}$	vs	SLA-DMB$_{KO}$-DMB-Myc$_{KI}$	-71,29	No	ns	>0,9999
SLA-DMA$_{KO}$	vs	SLA-DMB$_{KO}$-GFP	-113,9	No	ns	>0,9999
SLA-DMA$_{KO}$	vs	SLA-DMA$_{KO}$-DMA	-1630	Yes	****	<0,0001
SLA-DMA$_{KO}$	vs	SLA-DMA$_{KO}$-DMA-Myc$_{KI}$	-1437	Yes	****	<0,0001
SLA-DMA$_{KO}$	vs	SLA-DMA$_{KO}$-DMA-Strep$_{KI}$	-1597	Yes	****	<0,0001
SLA-DMB$_{KO}$	vs	SLA-DMB$_{KO}$-GFP$_{KI}$	-18,53	No	ns	>0,9999
SLA-DMB$_{KO}$	vs	SLA-DMB$_{KO}$-DMB$_{KI}$	-1605	Yes	****	<0,0001
SLA-DMB$_{KO}$	vs	SLA-DMB$_{KO}$-DMB-Myc$_{KI}$	-1085	Yes	****	<0,0001

KI: knockin; KO: knockout; vs: versus

II. Supplementary figures

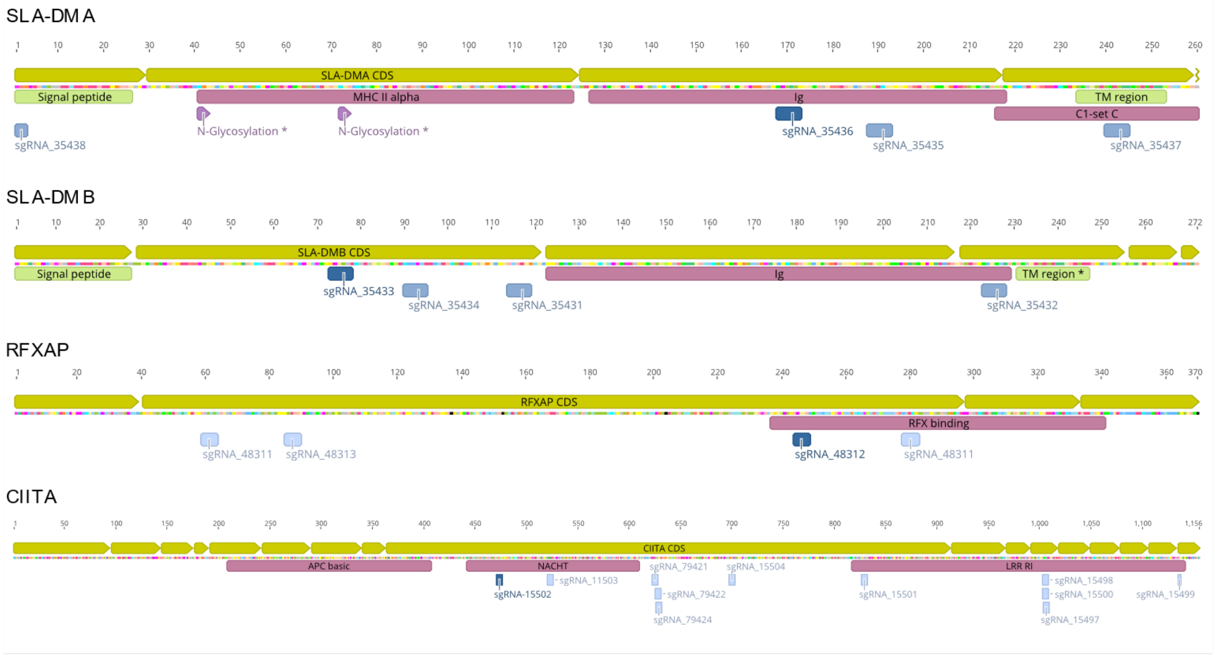

Figure S1. Schematic presentation of selected hits found by the genome-wide CRISPR/Cas9 knockout screen. Depicted are protein sequences deduced from the mRNAs of *SLA-DMA* (GenBank #NC_010449), *SLA-DMB* (GenBank #NC_010449), *RFXAP* (GenBank #NC_010453) and *CIITA* (GenBank #NC_010445, Isoform X4) which are composed of multiple exons (yellow arrows). Rectangles (light green, magenta) indicate relevant functional domains and post-translational modification sites. Only predicted features (Geneious Prime) are labeled by asterisks. Target regions of all library sgRNAs are shown in light blue, and the dark blue sgRNAs were selected for the generation of WSL_{KO} cells. Abbreviations: Ig, immunoglobulin domain; TM, transmembrane domain.

SLA-DMA

```
NC_010449                TGGGAGCATCACTCTGCTCCTGTG------------------------------GAAGGAATCGGG
WSL                      TGGGAGCATCACTCTGCTCCTGTG------------------------------GAAGGAATCGGG
WSL SLA-DMA_KO (11)      TGGGAGCATCACTCTGCTCCTGTG******************************GAAGGAATCGGG
WSL SLA-DMA_KO (12)      TGGGAGCATCACTCTGCTCCTGT-------------------------------GAAGGAGTCGGG
WSL SLA-DMA_KO (16)      TGGGAGCATCACTCTGCTCCTGT-------------------------------GAAGGAATCGGG

NC_010449                CCCACTTTCGTCTCCGCCACGGATGACCTCAGCTTCCAGGCCTTTTCTTATTTAAACTTCACACC
WSL                      CCCACTTTCGTCTCCGCCACGGATGACCTCAGCTTCCAGGCCTTTTCTTATTTAAACTTCACACC
WSL SLA-DMA_KO (11)      CCCACTTTCGTCTCCGCCACGGATGACCTCAGCTTCCAGGCCTTTTCTTATTTAAACTTCACACC
WSL SLA-DMA_KO (12)      CCCACTTTCGTCTCCGCCACGGATGACCTCAGCTTCCAGGCCTTTTCTTATTTAAACTTCACACC
WSL SLA-DMA_KO (16)      CCCACTTTCGTCTCCGCCACGGATGACCTCAGCTTCCAGGCCTTTTCTTATTTAAACTTCACACC
```

SLA-DMB

```
NC_010449                CAGGAGACCCGTATGGTCCCTTGT-GAATTTGGAGCGCTGAATGCGTTGGCCACATATTTCTCTG
WSL                      CAGGAGACCCGTATGGTCCCTTGT-GAATTTGGAGCGCTGAATGCGTTGGCCACATATTTCTCTG
WSL SLA-DMB_KO (9)       CAGGAGACCCGTATGGTCCCTTGT--AATTTGGAGCGCTGAATGCGTTGGCCACATATTTCTCTG
WSL SLA-DMB_KO (16)      CAGGAGACCCGTATGGTCCCTTGTTGAATTTGGAGCGCTGAATGCGTTGGCCACATATTTCTCTG
WSL SLA-DMB_KO (18)      CAGGAGACCCGTATGGTCCCTTGTTGAATTTGGAGCGCTGAATGCGTTGGCCACATATTTCTCTG

NC_010449                TTTACCTCAACCAGCAGGAAAAACTGCTCCAGCGCTTGTCCAATGGGCTCCAGAACTGTGCCACA
WSL                      TTTACCTCAACCAGCAGGAAAAACTGCTCCAGCGCTTGTCCAATGGGCTCCAGAACTGTGCCACA
WSL SLA-DMB_KO (9)       TTTACCTCAACCAGCAGGAAAAACTGCTCCAGCGCTTGTCCAATGGGCTCCAGAACTGTGCCACA
WSL SLA-DMB_KO (16)      TTTACCTCAACCAGCAGGAAAAACTGCTCCAGCGCTTGTCCAATGGGCTCCAGAACTGTGCCACA
WSL SLA-DMB_KO (18)      TTTACCTCAACCAGCAGGAAAAACTGCTCCAGCGCTTGTCCAATGGGCTCCAGAACTGTGCCACA
```

CIITA

```
NC_010445                AGCTGGGCCTGGGCTGACGGCCAGCTGCCACAGTACGACTTTGTCTTCTGCATCCCCTGCCACTG
WSL                      AGCTGGGCCTGGGCTGACGGCCAGCTGCCACAGTACGACTTTGTCTTCTGCATCCCCTGCCACTG
WSL CIITA_KO (1)         AGCTGGGCCTGGGCTGACGGCCAGCTGCCACA-TACGACTTTGTCTTCTGCATCCCCTGCCACTG
WSL CIITA_KO (4)         AGCTGGGCCTGGGCTGACGGCCAGCTGCC-----ACGACTTTGTCTTCTGCATCCCCTGCCACTG
WSL CIITA_KO (8)         AGCTGGGCCTGGGCTGACGGCCAG-----------------------CTGCATCCCCTGCCACTG

NC_010445                TTTGGACCGGCCGGGGAACACCTACCGCCTGCAGGATCTGCTCTTCTCCCTGGGCCCACAGCCCC
WSL                      TTTGGACCGGCCGGGGAACACCTACCGCCTGCAGGATCTGCTCTTCTCCCTGGGCCCACAGCCCC
WSL CIITA_KO (1)         TTTGGACCGGCCGGGGAACACCTACCGCCTGCAGGATCTGCTCTTCTCCCTGGGCCCACAGCCCC
WSL CIITA_KO (4)         TTTGGACCGGCCGGGGAACACCTACCGCCTGCAGGATCTGCTCTTCTCCCTGGGCCCACAGCCCC
WSL CIITA_KO (8)         TTTGGACCGGCCGGGGAACACCTACCGCCTGCAGGATCTGCTCTTCTCCCTGGGCCCGCAGCCCC
```

RFXAP

```
NC_010453                GCAGTGAGACCACAAG------------------------CCAGGTGGCCAAGCAGCGCAAGCCCT
WSL                      GCAGTGAGACCACAAG------------------------CCAGGTGGCCAAGCAGCGCAAGCCCT
WSL_RFXAP_KO (6)         GCAGTGAGACCACAAG-----------------------CCCAGGTGGCCAAGCAGCGCAAGCCCT
WSL_RFXAP_KO (8)         GCAGTGAGACCACAAG*************************CAGGTGGCCAAGCAGCGCAAGCCCT

NC_010453                GGATGTGCAAGAAGCACCGCAACAAGATGTACAAGGACAAGTACAAAAAGAAGAAAAGTGACCAG
WSL                      GGATGTGCAAGAAGCACCGCAACAAGATGTACAAGGACAAGTACAAAAAGAAGAAAAGTGACCAG
WSL_RFXAP_KO (6)         GGATGTGCAAGAAGCACCGCAACAAGATGTACAAGGACAAGTACAAAAAGAAGAAAAGTGACCAG
WSL_RFXAP_KO (8)         GGATGTGCAAGAAGCACCGCAACAAGATGTACAAGGACAAGTACAAAAAGAAGAAAAGTGACCAG
```

Figure S2. Characterization of WSL gene knockout cell clones. The sgRNA targeted gene regions of *SLA-DMA, SLA-DMB, RFXAP,* and *CIITA*, respectively, were amplified by PCR, sequenced, and analyzed for insertions or deletions (INDELs) of nucleotides compared to the GenBank reference sequences (bold) and the sequences of parental WSL cells. Asterisks indicate transfer vector sequence insertions of 823 nt in SLA-DMA_{KO} (11) and 140 nt in WSL $RFXAP_{KO}$ (8). Inserted (blue) and deleted (yellow) nucleotides are highlighted. Stop codons within the insertions or resulting from frameshifts are underlined. The premature termination codons of the WSL $CIITA_{KO}$ clones are located downstream of the shown sequence section.

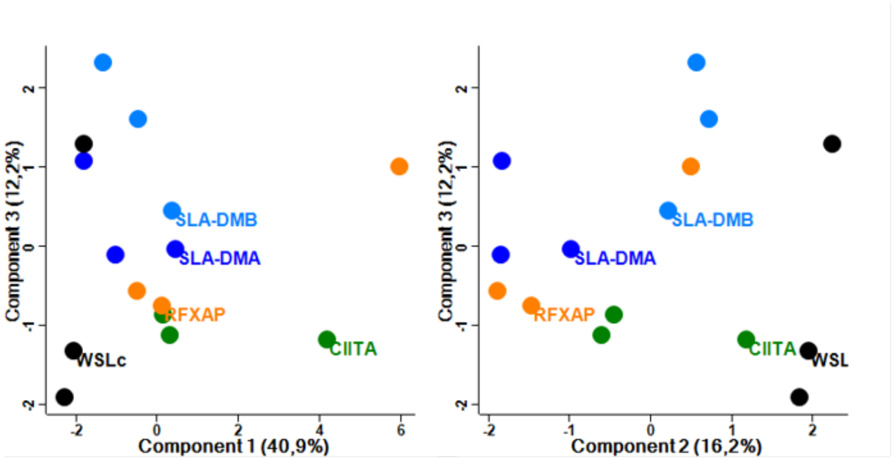

Figure S3. Principal component analysis of WSL and WSL knockout proteomes. The analysis is based on 4874 commonly expressed proteins and was performed in Perseus software. No marked clustering of the cell clones was observed indicating a high level of homogeneity between all samples.

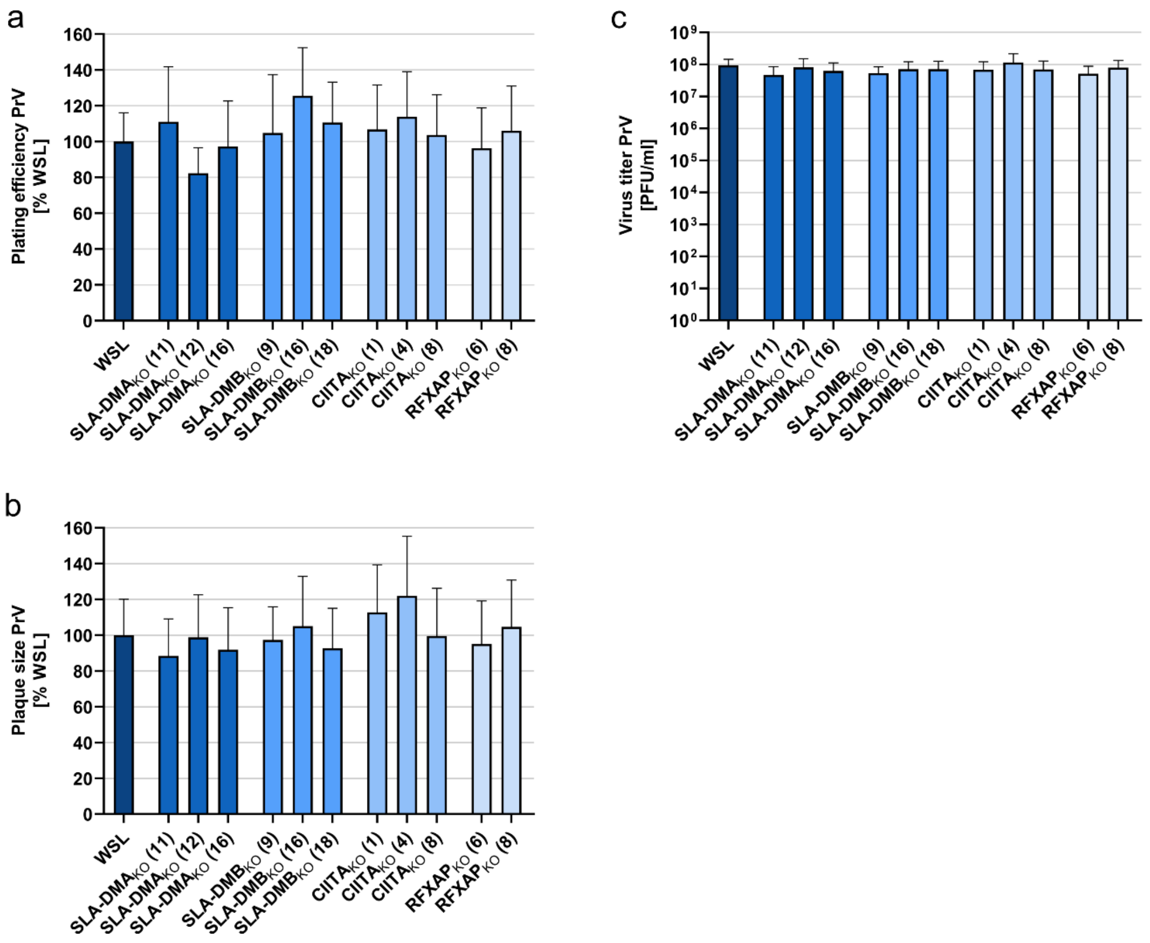

Figure S4. Growth properties of PrV in WSL knockout cells. (a, b) WSL and indicated WSL_{KO} cells were infected with serial dilutions of PrV-BaΔgGG, and incubated under semi-solid methocel medium for three days. **(a)** Plating efficiency was calculated based on counted plaques from four independent experiments. Shown are the relative (%) apparent titers compared to WSL cells with standard deviations. **(b)** Areas of approx. fifty plaques were measured in four independent experiments (n ≥ 167). Shown are the mean relative sizes with standard deviations. **(c)** WSL and WSL_{KO} cells were infected with PrV-BaΔgGG at a MOI of 0.02 and frozen after three days. Progeny virus titers were determined by standard plaque assay on RK13 cells. Shown are the mean virus titers (PFU/ml) of three independent experiments with two replicates each (n = 6) with standard deviations.

SLA-DMA CDS

```
ATGGACCACGAGCTGTCCCAGGGCGCCGCTTTGCTGCGCTTGCTGCATTTGCTGTGGCTT
CTGCCTCACAGTTGGACGGCACCAGAAGCGCCGGCGCCCGGATGGAGGGATGAGCTGCAG
AACCATACTTTCCAGTATACCATGTACTGCCAGGACGGTAATCCTGAGGTCGGCCTCAGT
GAGGTGTATGACGGCGATCAGCTGTTCTCATTTAATTTCTCCCAGAACATTAGAGTGCCT
CGCCTGCCGGAGTTTGCTGATTGGGCCCACCAGATTGAGGATACACCCGCGATCTTCTTT
GACAAGGGCTTCTGCCGCGAGATGATTGAGAAGGTGGGCCCACTGTTCGAGGGCAAGATT
CCCGTATCTCGCGGCCTCCCAATCGCAGAAGTCTTTACCCTCAAGCCGCTGGAGTTCGGC
AAGCCCAATACCTTGGTGTGCTTCGTCTCAAACCTGTTCCCACCCGCCCTGACTGTCACA
TGGGAGCACCATTCCGCTCCTGTCGAGGGCATTGGACCAACCTTTGTCTCTGCCACCGAT
GACTTGTCCTTTCAGGCATTCTCCTACCTGAATTTCACTCCTACACCAAGTGACCTGTTC
TCCTGCGTCGTGACCCACGAGCTCGACGGCTACGTCGCTATCTCTTACTGGGTCCCTCAG
AACGCCTTGCCATCCGACTTGCTCGAGAACGTACTCTGTGGTGTGGCGTTCGGTCTCGGA
GTCCTCGGCATTATCGTCGGATTGGTTCTGATTATCTATAGCAGGAAGCCTTGCTCAGCC
TGA
```

SLA-DMB CDS

```
ATGCCATGCATCAGTCCGGAGCAGAGCATGAGCGCTCTTCTGCAGCTCCTGCTCGGCTTG
AGCCTCGGATGTACCGGCGCTGGAGGCTTCGTCGCTCACGTGGAGTCTACCTGCCTGCTT
GATGACGAAGGCACTCCACAGGACTTCACATATTGCATCTCCTTCAACAAGGATCTGCTT
ACCTGTTGGGACCCACAGGAGACACGTATGGTGCCCTGCGAGTTCGGCGCTCTCAACGCG
CTGGCTACATACTTCTCCGTGTATCTGAATCAACAAGAGAAGCTCCTGCAGCGCTTGTCT
AACGGCCTCCAGAACTGCGCCACACATACTCAACCGTTTTGGAAGAGCCTCACTCATCGT
ACTCAGCCGCCATCCGTGCAGGTGGCCAAGACCACGCCGTTCAACACACGCGAGAGCGTC
ATGCTGGCTTGCTACGTGTGGGGCTTCTATCCGGCTGACGTCATTATCACCTGGCGCAAG
AACGGCCAGCCAGTGCTGCCTCACGGCAAGGCGCACATGATCACACAGCCTAACGGCGAT
TGGACTTATCAGACAGTGAGCCACCTGGCGACTACACCATCCTATGGAGATACCTACACC
TGCGTTGTGGAGCACATTGGCGTACCTGAACCTATCCTGCAGGACTGGACCTCCGGCCTG
TCACCTGTCCAAACCGTCAAGATCAGTGTTAGCGTGGCGACTCTCGGCCTTGGCCTGATT
ATCTTCAGCCTCGGCCTCCTGTCCTGTCAACGCTCAGTGGCTCCAGGTTACATCTTCCTG
CCTGGCACCACTTATCCAGAAGGCCAGCACATCTCCTGA
```

Figure S5. Synthetic open reading frames of SLA-DMA and SLA-DMB. Coding sequences of SLA-DMA (GenBank #NC_010449.5, nt 25133494 to 25137928) and SLA-DMB (GenBank #NC_010449.5, nt 25119278 to 25125089) were spliced in silico, codon optimized, and the binding regions of the selected sgRNA altered as far as possible by silent base substitutions. Custom made plasmids containing the open reading frames were produced synthetically (Invitrogen, Thermo Fisher Scientific).

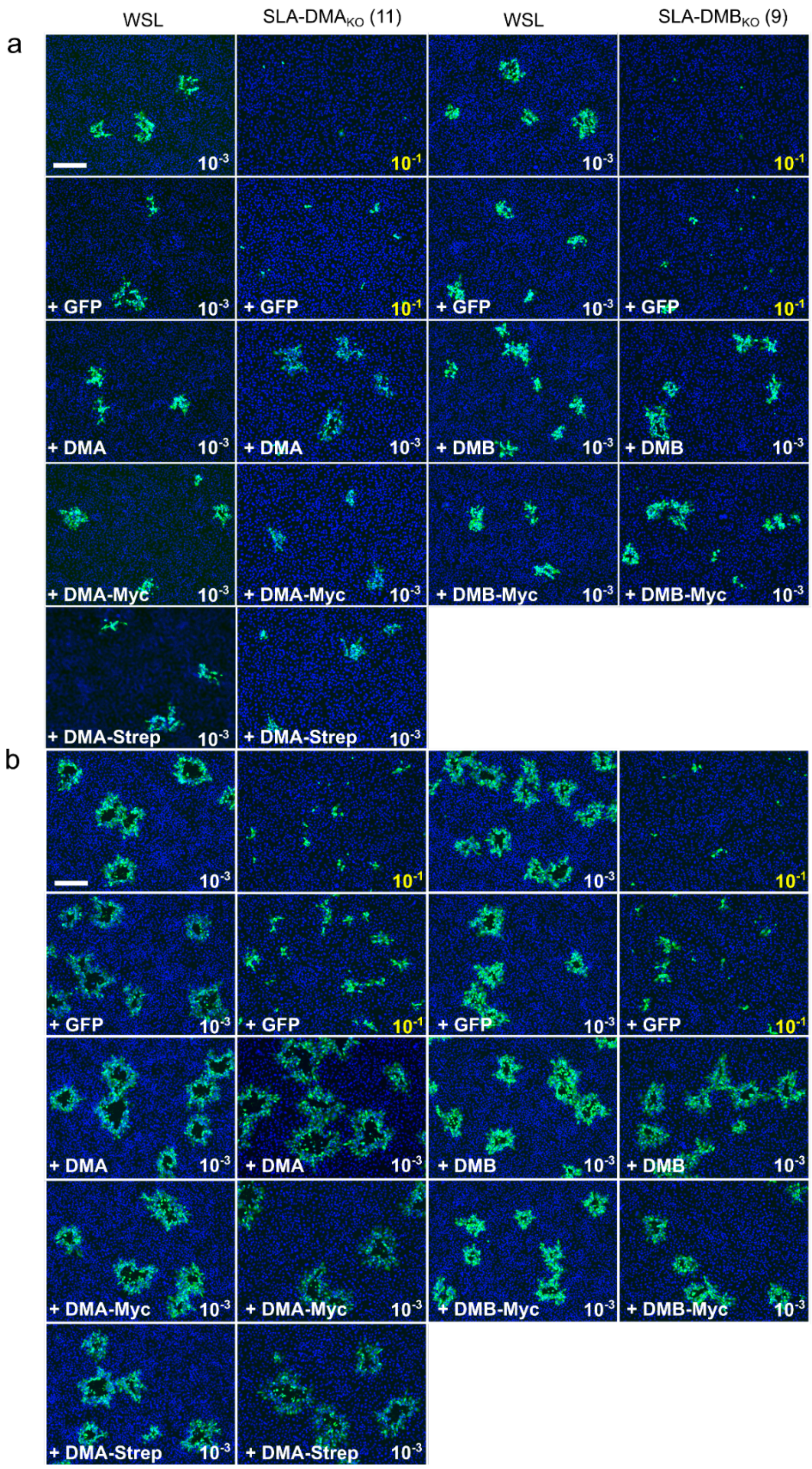

Figure S6. ASFV plaque sizes on WSL, WSL knockout and WSL knockout / knockin cells. WSL, WSL SLA-DMA$_{KO}$ and WSL SLA-DMB$_{KO}$ cell clones were stably lentivirus-transduced with expression cassettes for the indicated transgenes, and infected with 10^{-1} or 10^{-3} diluted **(a)** ASFV Armenia or **(b)** ASFV Kenya stocks. ASFV-infected cells (green) and DNA (blue) were visualized by (immuno-) fluorescence staining. Bar: 200 µm.

III. Original immunoblots

Original immunoblot for Fig. 6a

Immunoblot images with cropped areas indicated by dashed lines. Each membrane was incubated with specific antibodies indicated at the bottom of each picture.

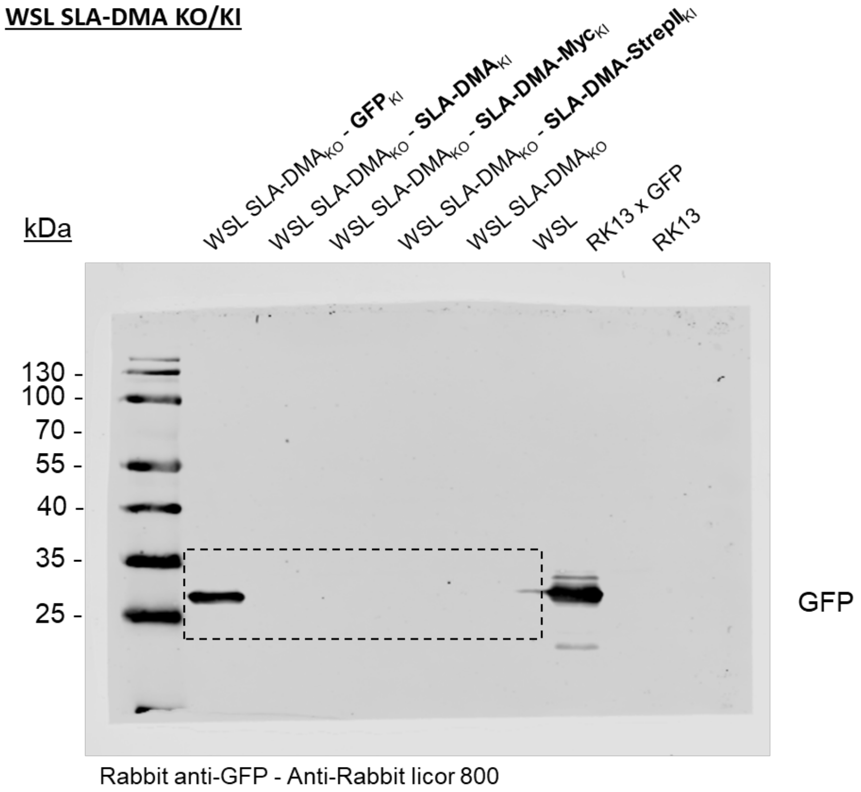

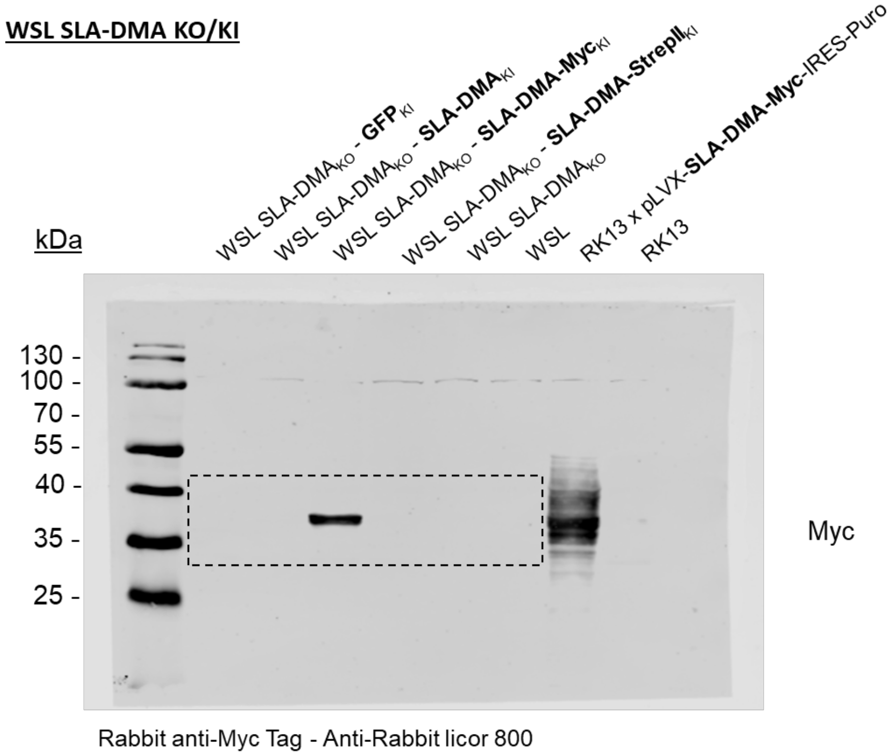

WSL SLA-DMA KO/KI

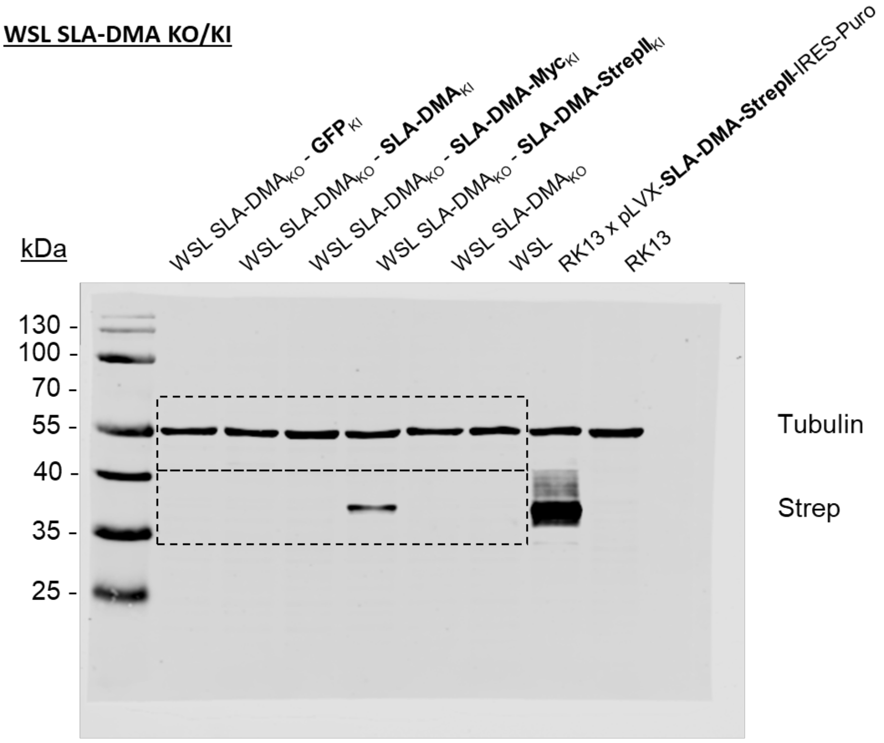

Rabbit anti-Strep Tag - Anti-Rabbit licor 800
Mouse anti-Tubulin - Anti-Mouse licor 680

Original immunoblot for Fig. 6b

Immunoblot images with cropped areas indicated by dashed lines. Each membrane was incubated with specific antibodies indicated at the bottom of each picture.

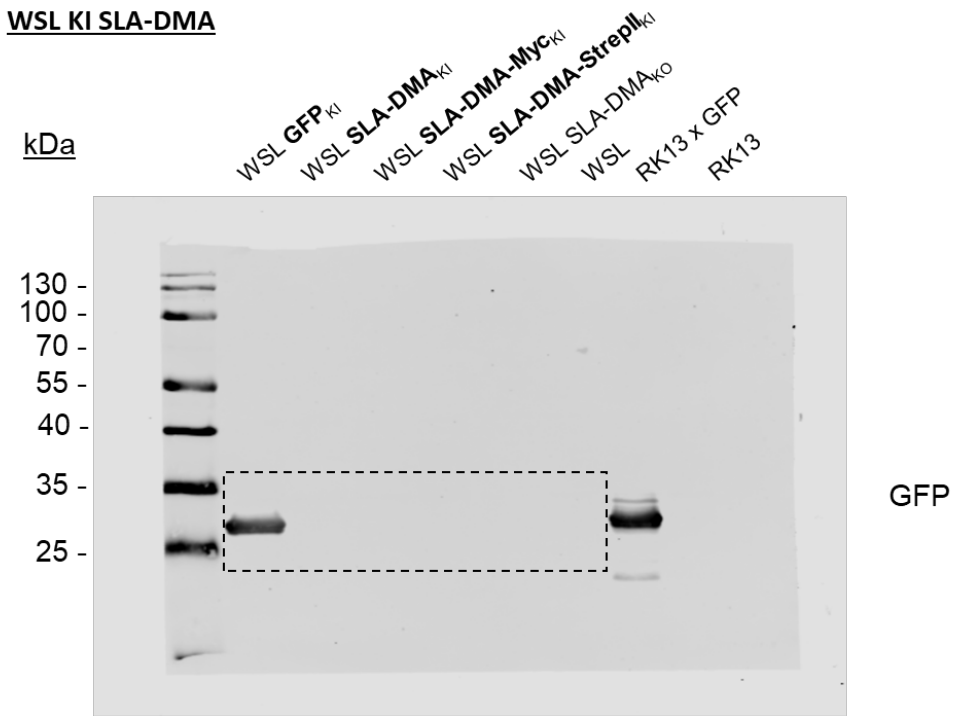

Rabbit anti-GFP - Anti-Rabbit licor 800

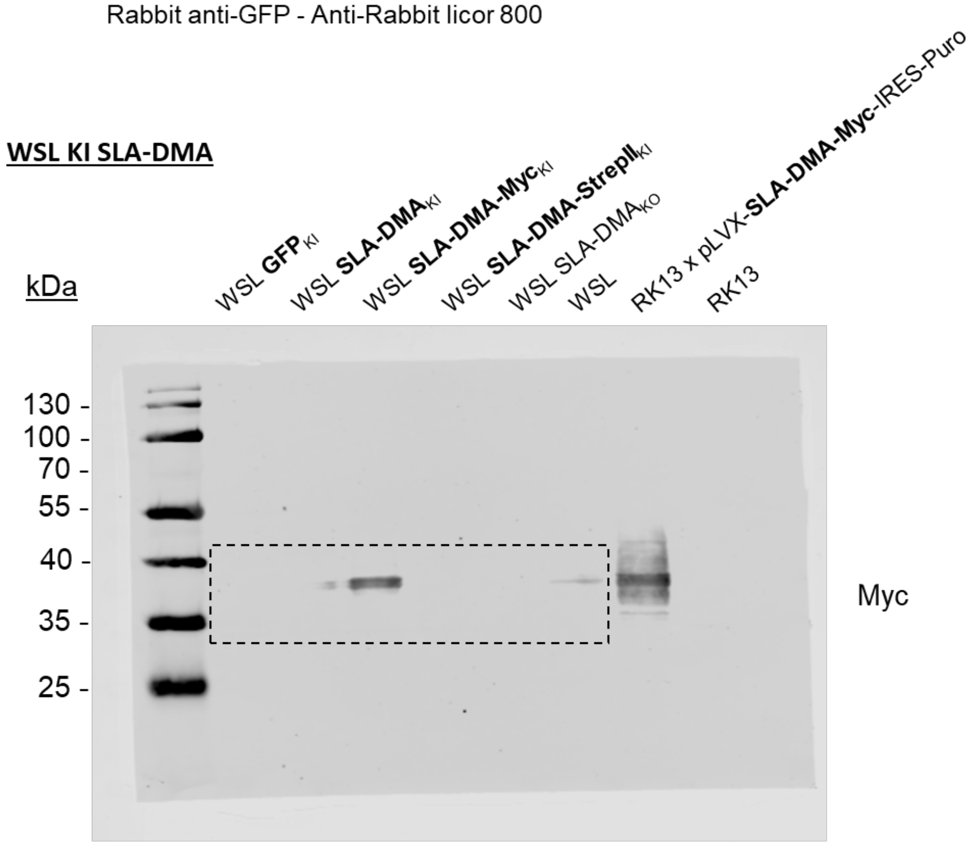

Rabbit anti-Myc Tag - Anti-Rabbit licor 800

WSL KI SLA-DMA

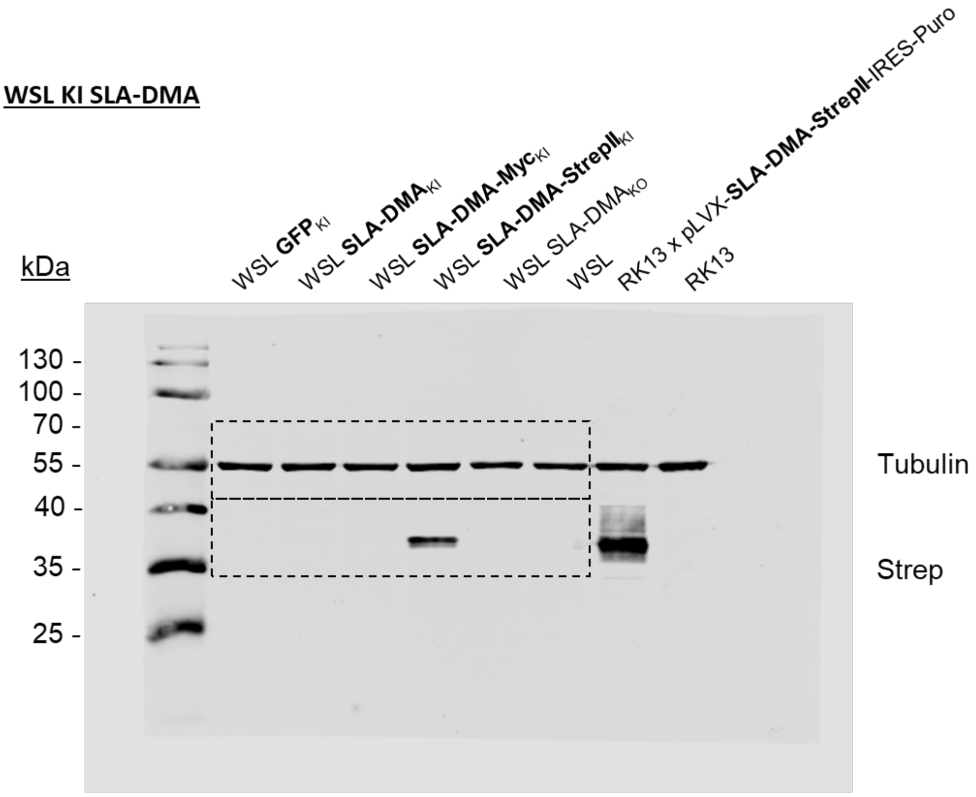

Rabbit anti-Strep Tag - Anti-Rabbit licor 800
Mouse anti-Tubulin - Anti-Mouse licor 680

Original immunoblot for Fig. 6c

Immunoblot images with cropped areas indicated by dashed lines. Each membrane was incubated with specific antibodies indicated at the bottom of each picture

WSL SLA-DMB KO/KI

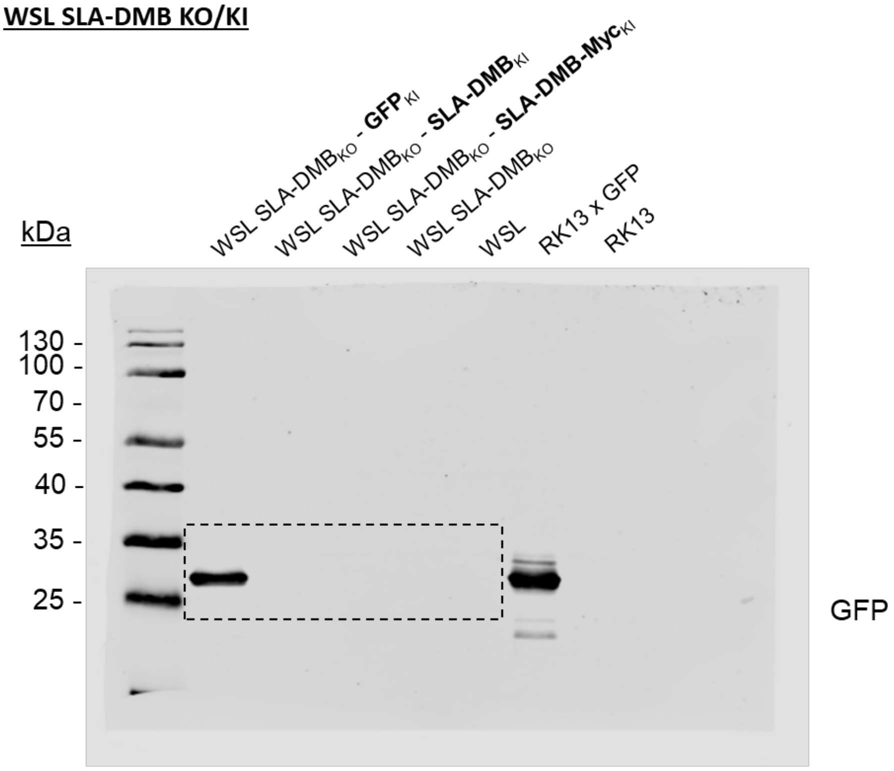

Rabbit anti-GFP - Anti-Rabbit licor 800

WSL SLA-DMB KO/KI

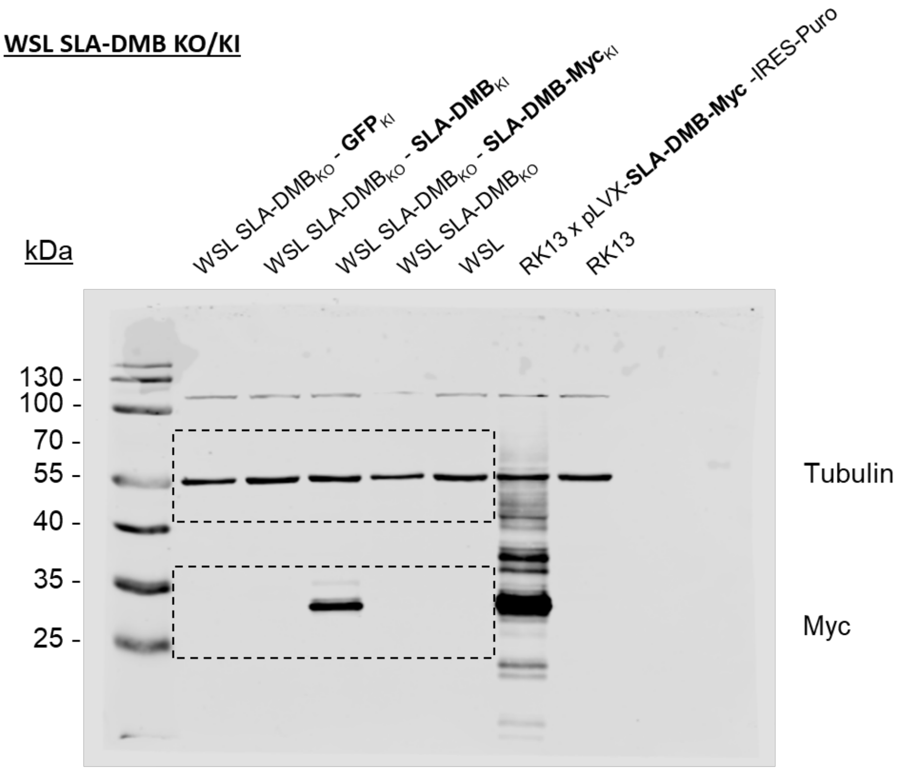

Rabbit anti-Myc Tag - Anti-Rabbit licor 800
Mouse anti-Tubulin - Anti-Mouse licor 680

Original immunoblot for Fig. 6d

Immunoblot images with cropped areas indicated by dashed lines. Each membrane was incubated with specific antibodies indicated at the bottom of each picture

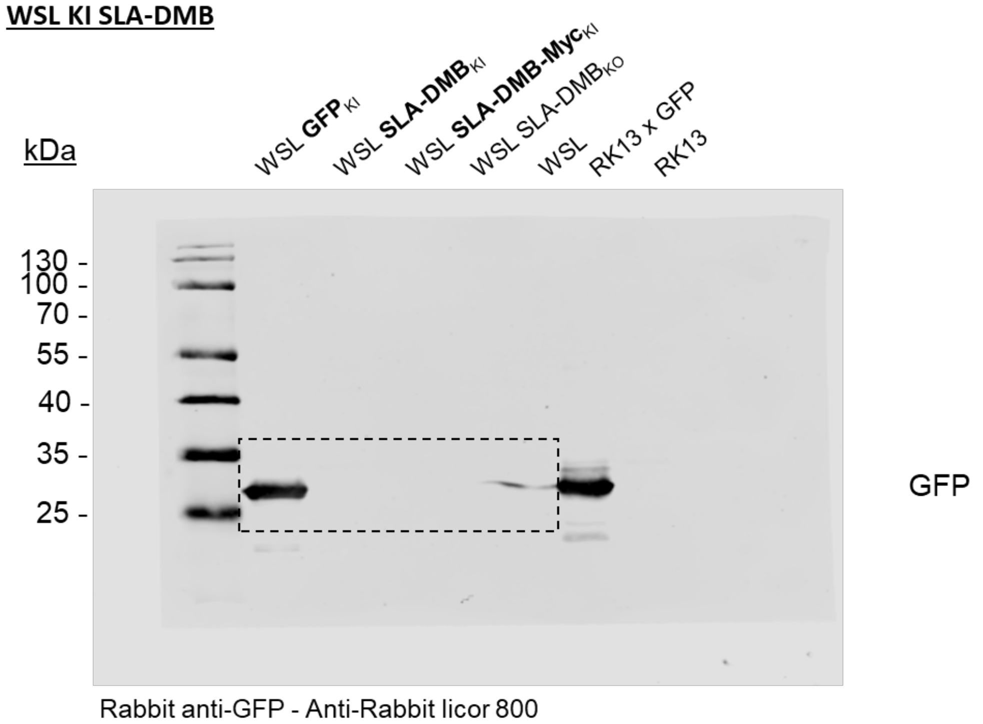

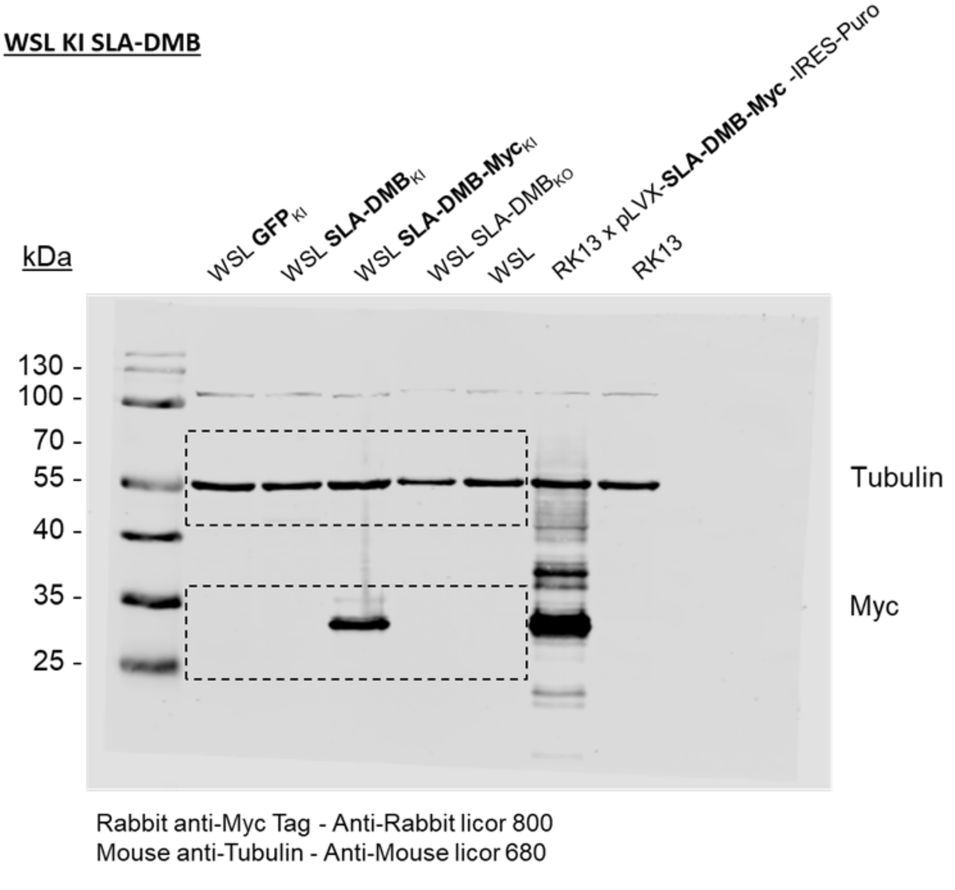

IV. Supplementary references

1 Li, W. *et al.* MAGeCK enables robust identification of essential genes from genome-scale CRISPR/Cas9 knockout screens. *Genome Biol.* **15**, 554, doi:10.1186/s13059-014-0554-4 (2014).
